# Recent development of polymer nanomicelles in the treatment of eye diseases

**DOI:** 10.3389/fbioe.2023.1246974

**Published:** 2023-08-04

**Authors:** Ruijun Cai, Ling Zhang, Hao Chi

**Affiliations:** ^1^ Department of Pharmacy, The People’s Hospital of Jiuquan, Jiuquan, Gansu, China; ^2^ Qingdao Municipal Hospital, University of Health and Rehabilitation Sciences, Qingdao, China

**Keywords:** eye diseases, polymer nanomicelles, treatment, drugs, biological barriers

## Abstract

The eye, being one of the most intricate organs in the human body, hosts numerous anatomical barriers and clearance mechanisms. This highlights the importance of devising a secure and efficacious ocular medication delivery system. Over the past several decades, advancements have been made in the development of a nano-delivery platform based on polymeric micelles. These advancements encompass diverse innovations such as poloxamer, chitosan, hydrogel-encapsulated micelles, and contact lenses embedded with micelles. Such technological evolutions allow for sustained medication retention and facilitate enhanced permeation within the eye, thereby standing as the avant-garde in ocular medication technology. This review provides a comprehensive consolidation of ocular medications predicated on polymer nanomicelles from 2014 to 2023. Additionally, it explores the challenges they pose in clinical applications, a discussion intended to aid the design of future clinical research concerning ocular medication delivery formulations.

## 1 Introduction

Eye diseases present substantial threats to health and wellbeing, currently ranking as the third most prevalent category of illness following cancer and cardiovascular diseases (Zhao, Wang, and Du, 2021). The modern societal landscape, characterized by extended screen usage, substantial radiation exposure, and environmental pollutants, inadvertently burdens our ocular health. Consequently, the incidence rates of a range of eye diseases such as corneal injuries, macular degeneration, cataracts, glaucoma, diabetic retinopathy, and eye infections, are escalating. The gravity of these afflictions is further underscored by their potential to culminate in vision loss in severe cases (Xu et al., 2020), thus necessitating the advancement of research on eye diseases and associated treatments. Topical administration, inclusive of eye drops, eye ointments, and eye gels, serves as the treatment modality for an estimated 90% of eye diseases (Lim et al., 2016; Rodríguez et al., 2016; Upadhayay, Kumar, and Pathak, 2016; Xu et al., 2016).

Nonetheless, the presence of several biological barriers within the eye impedes the bioavailability of many medications, consequently impacting their therapeutic efficacy. This hurdle necessitates the administration of frequent, high-dosage treatments that potentially lead to undesirable side effects and diminished patient tolerance (Li, Zhang, et al., 2012; Liu, Jones, and Gu, 2012). Such limitations significantly impede the treatment efficacy of medications for eye diseases, posing a considerable challenge to the development of safe and effective ocular medication delivery technology.

Existing research indicates the substantial potential of polymer nanomicelles owing to their unique characteristics such as mucosal adhesion and small size. These qualities can enhance the bioavailability of medications, facilitate superior corneal penetration and intraocular absorption, decrease eye irritation, and minimize adverse medication reactions (Rupenthal, Green, and Alany, 2011; Enriquez et al., 2013; Kwon et al., 2013; Xu et al., 2013; Franca et al., 2014; Ashley et al., 2016; Salatin et al., 2016; Shirizadeh et al., 2017; Salatin, 2018). Polymeric micelles (PMs), the most successful among the nano-carrier systems, can be readily produced via self-assembly. They also offer flexibility in modulating their physical, chemical, and surface properties through modifications in copolymer structure or surface adjustments (Hwang, Ramsey, and Kabanov 2020; Yu et al., 2020; Kaur et al., 2021; Ghezzi et al., 2022).

A multitude of polymeric micelle-based medication delivery formulations have now advanced to various stages of clinical and preclinical trials across different nations (Anselmo and Mitragotri, 2016; 2019; Germain et al., 2020; Zheng et al., 2021). Over the past decade, notable developments include the authorization of treatments such as rapamycin nano-micelle eye drops for immune rejection inhibition, terbinafine hydrochloride nano-micelle preparations for combatting ocular fungal infections, and Cequa^®^ (a 0.09% cyclosporine eye solution) for dry eye treatment. Despite these advancements, the number of related formulations that have been successfully marketed and incorporated into clinical practice remains limited.

This paper seeks to review the recent advancements in the application of polymer nanomicelle dosage forms in eye disease treatment over the last decade.

## 2 The structure of the eye and the delivery of eye medication

Anatomically, the eye can be delineated into two segments: the anterior and posterior segments. Constituents of the anterior segment encompass the cornea, conjunctiva, aqueous humor, iris, ciliary body, and lens, whereas the posterior segment is formed by the sclera, choroid, and retinal pigment epithelium. The distinct architecture of the eye serves a dual purpose: protection against pathogenic intrusions and unfortunately, posing as a formidable barrier to medication penetration. Consequently, the anterior and posterior segments of the eye present unique challenges in medication administration.

The primary impediments to ocular drug delivery comprise of the conjunctiva, tear film, cornea, blood-aqueous barrier, and blood-retinal barrier, with the cornea and retina proving particularly resistant to drug penetration. The structural complexity of the cornea, divided into the external epithelium, the medial stroma, and the internal endothelium, compounds this resistance. The corneal epithelium, possessing hydrophobic a characteristics, acts as a permeation obstacle for hydrophilic drug formulations. Conversely, the stroma, constituting 90% of the corneal volume, exhibits strong hydrophilic tendencies, which restrict the penetration of hydrophobic drugs. The innermost endothelium, however, functions as a hydrophobic barrier. Thus, the effective transcorneal permeation rates of drugs are influenced by their individual hydrophobic and hydrophilic properties.

The blood-aqueous barrier is primarily composed of endothelial cells residing in the anterior uvea. This structure restricts the ingress of hydrophilic drugs into the anterior chamber from the plasma. Uniquely positioned within the eye, the blood-retinal barrier consists of two layers. The outer layer is situated on the retinal pigment epithelial cells, and the inner layer is located at the tight junctions of the retinal capillaries. This configuration impedes the entry of water-soluble molecules into the retina (Yu et al., 2022). The cornea and conjunctiva harbor numerous transporters implicated in medication transport across the ocular surface. Among these, P-glycoprotein (P-gp) and multi-drug resistance protein (MRPs) function as efflux transporters, thus limiting medication uptake.

**FIGURE 1:**
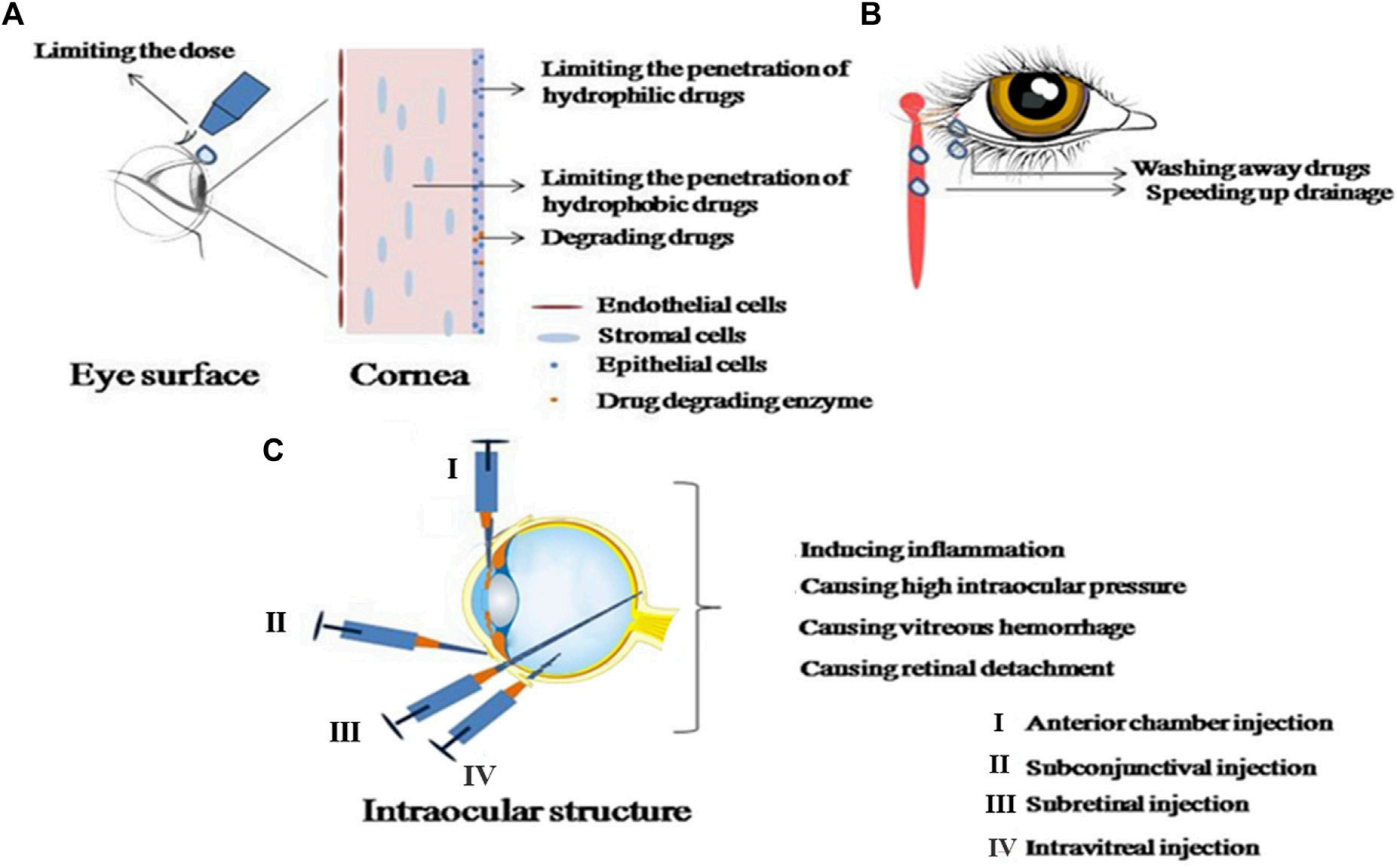
Details the obstacles and complications in ophthalmic administration. **(A)** Represents the obstacles in ophthalmic administration; **(B)** shows the removal mechanisms of ophthalmic administration; **(C)** illustrates the complications associated with intraocular injection.

Ocular medication delivery primarily serves as the treatment for local diseases and can be categorized into local, systemic, periorbital, and intravitreal delivery (refer to Figure 1). Compared to systemic delivery, the former offers enhanced medication enrichment at therapeutic targets and can minimize adverse reactions associated with systemic delivery. For anterior eye diseases, therapeutic medications operate through the cornea, conjunctival epithelium, or the blood-aqueous barrier. In contrast, therapeutic medications for posterior eye diseases must traverse multiple internal barriers, including the lens, vitreous, or blood-retinal barrier, during non-invasive local administration. This necessitates repeated invasive procedures such as local vitreal or subconjunctival injections for treating posterior eye diseases (Lakhani, Patil, and Majumdar, 2018). Invasive methods of drug administration not only induce patient discomfort during the procedure, but their long-term application can also precipitate secondary ocular inflammation and an increase in intraocular pressure (Kim et al., 2020). Ocular injections, for example, may result in complications such as pain, ocular inflammation, intraocular hemorrhage, elevated ocular pressure, and retinal detachment (refer to Figure 1). Procedures involving physical intrusion carry a higher risk of inducing infections. During injection-based treatments, if the needle inadvertently punctures a blood vessel, it can trigger severe hemorrhaging. Vitreous injections can similarly contribute to heightened intraocular pressure. These adverse reactions can be attributed to mechanisms such as transient inflammation triggered by the production of intraocular pressure-inducing cytokines, and mechanical blockage of the trabecular meshwork. (Ricca, Morshedi, and Wirostko, 2015). Common local ophthalmic formulations, like ordinary eye drops, tend to overflow or drain into the nasolacrimal duct upon application to the eye surface. This, coupled with the dilution effect of tears, significantly reduces medication bioavailability. Furthermore, regular eye drops can induce tearing and tear film renewal, causing most medications to be washed away within 15–30 s. Consequently, the contact time between medications and the ocular surface is short, resulting in low bioavailability. Therefore, it is crucial to enhance the medication’s residence time on the eye surface and promote corneal permeability. While the hydrophilic corneal stroma primarily facilitates the diffusion of hydrophilic components, the delivery of many therapeutically significant hydrophobic medications is compromised (Occhiutto et al., 2012; Maulvi, Soni, and Shah, 2016).

The challenges presented by ocular medication delivery are significant in the treatment of eye diseases. In response, recent years have witnessed a concentrated effort from researchers to develop novel ocular medication delivery systems. These systems aim to possess a prolonged residence time and the ability for sustained, controlled release, thus extending the duration of action, reducing systemic absorption, mitigating transporter effects, and maximizing medication bioavailability. To surmount the barriers posed by the cornea and conjunctiva in ocular medication delivery, research on polymer nanomicelles in the treatment of eye diseases has seen a yearly upsurge.

## 3 Introduction of the characteristics of polymer nanomicelles

Polymer nanomicelles are core-shell micelles that form spontaneously due to intermolecular hydrogen bonding, electrostatic interactions, and van der Waals forces. This formation occurs among amphiphilic polymers. Numerous water-insoluble medications, encompassing proteins and gene-based medications, can be enveloped within the hydrophobic core during micelle core formation. When delivering medication, polymer nanomicelles maintain commendable pharmacokinetic characteristics, pharmacological traits, and medication stability, thereby enhancing the effectiveness of disease treatment (Li, Liu, Ke, et al., 2021).

Relative to small-molecule surfactants, amphiphilic polymers exhibit a lower critical micelle concentration (CMC), which confers increased resistance to solution dilution in polymer nanomicelles. The size of these micelles usually ranges between 10–100 nm. This nanometer range favors cellular uptake of micelles, allowing for medication delivery via endocytosis, and thereby circumventing multi-drug resistance resulting from medication release mechanisms (Ke et al., 2014). Given the variability in micellar core-shell structures, polymers can freely select appropriate carriers based on the embedded medication’s properties, action sites, administration routes, and pathophysiological conditions.

### 3.1 Polymer nanomicelles support materials

#### 3.1.1 Natural polymer nanomicelles carrier materials

Carrier materials for polymer nanomicelles, contingent upon their origin, can be bifurcated into two categories: natural and synthetic. The ubiquity of natural high polymer materials, coupled with their superior biocompatibility and the non-toxic nature of their degradation products in the human body, makes them appealing for chemical modifications. These modifications can bestow these polymers with novel functional groups, making this area a focal point of current research. The repertoire of natural polymer nanomicelles carrier materials encompasses hyaluronic acid, albumin, and chitosan.

Chitosan, derived from chitin through deacetylation, is distinguished as the sole alkaline polysaccharide among natural polysaccharides. It also holds the distinction of being the most thoroughly studied and utilized high polymer material in natural polymer micelles, particularly in the field of ocular drug delivery. Its ability to amplify the permeability of corneal epithelial cells can enhance the performance of nano-micelles (Grimaudo et al., 2019). Chitosan’s solubility in aqueous solutions is confined to acidic environments, wherein it can undergo a solution-gel transition when pH exceeds 6.2. Given the slightly acidic pH of tears, which falls in the 6.5–7.60 range, chitosan can function as a pH-responsive material, extensively harnessed for *in-situ* gel formation in ophthalmic drug delivery.

Hyaluronic acid (HA), characterized by exceptional biocompatibility, biodegradability, bioadhesiveness, viscoelasticity, and receptor interaction attributes, can serve as an effective drug carrier for the treatment of ocular diseases. HA’s mucosal adhesion potential can augment the corneal adhesion of the carrier, thus raising the prospects of corneal penetration and improving drug utilization (Zhang et al., 2021). Beyond its local application, HA’s tumor-targeting capabilities can be leveraged for the treatment of ocular tumors (Li et al., 2020). The encapsulation of hydrophobic drugs within polymer micelles displaying high anti-inflammatory capacity, followed by their amalgamation with hyaluronic acid (HA), can enhance the anti-inflammatory response and facilitate efficient drug delivery (Shi et al., 2023). When contrasted with conventional, commercially available eye drops, the application of albumin nanoparticles, carrying anti-inflammatory piroxicam, into rabbit eyes elevated the bioavailability by a factor of 1.8 (Giunchedi et al., 2000). Albumin has also been merged with chitosan to generate drug formulations laden with anesthetics such as tetracaine and atropine (Addo et al., 2015). Microencapsulated tetracaine and atropine appear to significantly extend the duration and apex of drug action, compared to standard drug solutions.

#### 3.1.2 Synthetic polymer nanomicelles carrier materials

Biocompatible copolymers such as polyethylene glycol (PEG), polyethylene oxide (PEO), polyacrylamide (PAM), polyvinylpyrrolidone (PVP), polyvinyl alcohol (PVA), polyethyleneimine (PEI), and complexes formed by PEO and polyelectrolytes constitute the hydrophilic sections of polymer nanomicelles. Polyethylene glycol (PEG), particularly within the molecular weight range of 1,000–12000 Da, is the most commonly employed hydrophilic segment. PEG within this range offers excellent water solubility, non-toxicity or low toxicity, and non-immunogenicity (Lavasanifar, Samuel, and Kwon, 2002). Polyethylene glycol (PEG) and its derivatives hold significant value in the biomedical field. For instance, DMG-PEG2000 is a critical component of the COVID-19 vaccine liposome delivery system (Buschmann et al., 2021). Among potential alternatives to PEG, poly (N-vinyl-2-pyrrolidone) is often considered the primary substitute. However, some studies have shown that PEG-b-PLA micelles have potential toxicity by accumulating and upregulating pro-inflammatory genes and producing ROS (Dvořáková et al., 2017). Some studies also suggest that PEG-b-PLA micelles may interfere with the activation and function of the hypothalamic pituitary gonadal (HPG) axis, causing neuroendocrine disruptions (Dvořáková et al., 2017). Therefore, robust pharmacokinetic studies on polymeric micelles prepared using these carrier materials are needed for clinical treatments and research (Monika et al., 2017).

Within the realm of polymer nanomicelle drug delivery system, the degree of compatibility between the hydrophobic segment and the pharmaceutical molecule primarily dictates the efficiency of drug encapsulation. The interaction chemistry inherent to the hydrophobic constituents and pharmaceutical molecules, complemented by the dimensions of the hydrophobic chains, serves as additional determinants of the drug loading capacity. The diverse molecular attributes of pharmaceutical compounds underscore the reality that no single hydrophobic segment is capable of maximally encapsulating every class of medication. Moreover, the properties of the hydrophobic segment inherently affect the release dynamics of the encapsulated medications within the micellar structure. Commonly investigated hydrophobic components include biodegradable polyesters and amino acids, such as polylactide (PLLA), polyglycolide (PGA), polycaprolactone (PCL), polylactic glycolate (PLGA), polyaspartic acid (PAsp), polybenzylaspartic acid (PBLA), and polyglutamic acid (PGlu). Of these, PCL, PGA, and PLA have received FDA approval due to their exceptional biodegradability and compatibility. Certain hydrophobic block polymers, like poloxamer (polyethylene oxide, polypropylene oxide, polyethylene oxide), can alter ATP levels in cells, change the fluidity of the cell monolayer membrane, inhibit the exclusion of P-glycoprotein associated with multi-drug resistance, and resolve the issue of multi-drug resistance (Lavasanifar, Samuel, and Kwon, 2002, Monika et al., 2017). Poly (L-Lactide) (PLLA) is restricted in its clinical application due to its hydrophobicity, low degradation rate, and acidic degradation products (Liu et al., 2018; Caracciolo et al., 2021). However, the combination of PEG and PLLA exhibits excellent hydrophilicity and biocompatibility, making it a promising medication carrier matrix. Furthermore, introducing PEG can expedite the degradation of PLLA and decrease the acidity of degradation products.

### 3.2 Types of polymer nanomicelles

#### 3.2.1 Amphiphilic block copolymer

**FIGURE 2:**
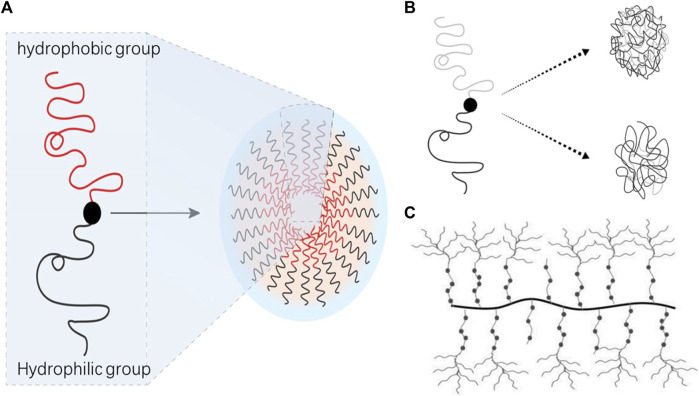
Types of polymer nanomicelles. **(A)** Amphiphilic block copolymer; **(B)** amphiphilic random copolymer; **(C)** graft polymer micellar.

Block copolymers consist of two or more distinct monomer units arranged sequentially, forming evident interfaces between different blocks (refer to Figure 2). This structural distinction gives block copolymers unique physical and chemical properties. *In vivo*, block polymer nanomicelles are less likely to be recognized as foreign bodies, and their hydrophilic shell helps evade detection by the endothelial network system, thereby reducing micelle exclusion from the bloodstream (Packhaeuser et al., 2004). When preparing polymer nanomicelles, it is essential to focus on achieving thermodynamic and kinetic stability. The thermodynamic stability of block polymer nanomicelles correlates with the critical micelle concentration of nanoparticles. This can be enhanced by minimizing the critical micelle concentration and strengthening the binding of hydrophobic nuclei in micelle nanoparticles. The hydrophilic-hydrophobic balance of amphiphilic block copolymers directly impacts the critical micelle concentration. Stability can be improved by adjusting this balance. Hydrogels fabricated from amphiphilic block polymers alleviate the necessity for surface treatment and offer dual benefits for the eyes, namely, providing hydration and thwarting surface deposition. Zhang et al. (Zhang et al., 2017) successfully synthesized a triblock copolymer, specifically poly (methyl methacrylate-vinyl acetate)-polyethylene glycol-poly (methyl methacrylate-vinyl acetate). This novel material, derived from the aforementioned triblock copolymer, is capable of significantly diminishing surface hydrophobicity and averting protein adsorption, thereby displaying a superior antifouling capacity. It can be effectively deployed for antifouling applications on the superior and inferior surfaces of ophthalmic devices. For Such attributes indicate its substantial potential for use in flexible contact lens manufacturing. This indicated increased micelle stability with the elongation of the hydrophobic part of the block copolymer. The properties of the hydrophobic part directly affect the critical micelle concentration, and chemical modifications can flexibly control nanoparticle stability. For instance, increasing the benzyl group content from 44% to 75% in the polyethylene glycol-b-benzyl protected polyaspartic acid amphiphilic block copolymer reduces the critical micelle concentration tenfold (Opanasopit et al., 2004), consequently enhancing copolymer stability. PLA-PEG, an amphiphilic block copolymer with high biocompatibility and biodegradability approved by the US Food and Medication Administration, has been studied for loading nifedipine into polymeric micelles, significantly improving medication solubility and bioavailability (Xu, Qiu, et al., 2019).

#### 3.2.2 Graft polymer micellar

Graft polymeric micelles, typically composed of amphiphilic graft polymers featuring hydrophobic backbone chains and hydrophilic branch chains, are gaining significant attention in the realm of polymer materials for injection due to their superior biocompatibility and biodegradability. These polymers, when dispersed in water, self-assemble into nanoparticles bearing a core-shell structure. The core of the micelle is formed by the hydrophobic skeleton, while the shell is constituted by the hydrophilic branch chains (refer to Figure 2).

One prominent example is Soluplus, an amphiphilic and biodegradable graft copolymer composed of polyethylene-caprolactam-polyvinyl acetate-polyethylene glycol (PVC-PVA-PEG). This copolymer is utilized to enhance the micellization or nanoparticle formation ability of insoluble medications, largely due to its low critical micelle concentration (CMC) (Zhang et al., 2020). Demonstrating superior eye tolerance and corneal permeability, Soluplus micelles have been effectively employed as topical ocular medication delivery systems for drugs that are poorly soluble in water (Mehra, Aqil, and Sultana, 2021).

#### 3.2.3 Amphiphilic random copolymer

Amphiphilic polymer micelles, due to their diverse microstructures, encompass random copolymers, block copolymers, and graft copolymers, representing three common polymer architectures. These primarily differ in their polymer chain arrangement methodologies. In random copolymers, disparate monomer units are randomly organized within the polymer chain, devoid of discernible block structures, typically exhibiting favorable solubility and fluidity characteristics (refer to Figure 2). Despite these qualities, the comprehensive research dedicated to the self-assembly of random copolymers significantly lags behind that of block and graft copolymers. Furthermore, research focused on drug loading utilizing amphiphilic random copolymers remains scarce. Poly (glycolide-co-L-lactide-co-caprolactone) (PGLC), a novel degradable high polymer material, has shown promise as it can self-degrade and be metabolized *in vivo*. Leveraging its unique characteristics, PGLC can serve as a sustained-release vehicle for certain drugs. Employing PGLC as the carrier, exploratory studies have been initiated to examine the release behavior of various drugs, including 5-fluorouracil. This research has led to the development of a novel intraocular implant immunosuppressant PGLC-cyclosporine drug release system. By directly implanting the PGLC-cyclosporine formulation into the eye, the blood-eye barrier can be effectively surmounted, yielding high intraocular drug concentrations. This method offers the benefits of low dosage requirements, long-term drug release, and carrier absorption within the body (Shi, Xie, and Wang, 2002).

#### 3.2.4 Polyelectrolyte micellar

Block or graft copolymers bearing charges can generate nanomicelles via electrostatic or hydrogen bonding with oppositely charged entities, such as polymers, DNA, polyelectrolytes, and enzymes. The aggregation process of polyelectrolyte micelles into a core structure is governed by intermolecular forces, encompassing hydrophobic interaction, electrostatic interaction, metal chelation, and hydrogen bonding among block copolymers. Traditional nanomicelle preparation methods predominantly involve the synthesis of covalently bonded block or graft amphiphilic polymers, which undergo self-assembly in water. This preparation process necessitates a substantial quantity of organic solvent, and the principal drug loading methodologies, namely, physical entrapment and adsorption, typically yield a relatively low loading rate for many ionic drugs. In recent years, there has been a shift towards using high polymer materials with excellent biological properties as drug carriers. Due to their charged cores, polyelectrolyte micelles can function as carriers for numerous charged drugs (such as DNA, RNA), thereby enhancing the stability of these types of drugs. This offers them a unique edge in gene delivery (Zhang et al., 2005). Given the large molecular chains of polyelectrolytes and their ionization characteristics reminiscent of small molecules, they can be employed in sustained and controlled drug delivery technologies. Presently, research concerning such micellar delivery systems in the context of ophthalmic diseases remains relatively nascent.

#### 3.2.5 Intelligent polymer nanomicelles delivery system

Beyond categorizations based on polymer structure, certain polymer nanomicelles carry distinctive structural groups with intelligent response capabilities, warranting classification based on response types. These so-called “smart” polymer nanomicelles delivery systems, or stimulus-responsive polymeric micelle delivery systems, are formed by stimulus-responsive polymers. These polymers exhibit changes in properties such as morphology, hydrophilicity and hydrophobicity, and permeation rate under the influence of specific environmental stimuli, including temperature, pH, ionic strength, and enzymatic activity. Some polymers not only respond to a single-factor stimulus but can react to two or more stimuli (Galaev and Mattiasson, 1999; Hoffman et al., 2000; Gupta, Vermani, and Garg, 2002; Kikuchi and Okano, 2002), allowing medications to release the required effective dose at the necessary time and specific location, achieving precision-controlled medication release. Multiple ocular medication delivery systems exist, including pH-responsive, temperature-responsive, and electrically responsive.

As a novel medication delivery system, pH-responsive polymer nanomicelles hold promising application potential due to their ability to actively target therapeutic sites. The pH value of tears ranges from 6.5 to 7.60, denoting weak acidity. Infections by fungi and bacteria alter the normal tissue and body fluid environment, resulting in acidic pH and heightened virulence factors (Wang et al., 2017). Research has shown that substances such as staphylococcus-secreted lactic acid or *E. coli* (*Escherichia coli*) can induce pH-triggered antibiotic release by local environment acidification (Hizal et al., 2015; Craig et al., 2016). Sukhishvili created an antibacterial multilayer film comprised of self-defensive tannic acid/cationic antibiotics (tobramycin, gentamicin sulfate, and polymyxin B), which releases antibacterial medications in response to bacterial infection-induced environmental acidification (Zhuk et al., 2014). Two synthesis strategies exist for pH-sensitive polymer nanomicelles: one hinges on protonation or deprotonation of acid-sensitive bonds for sustained, controlled medication release, the other relies on the use of acid-sensitive bonds as connecting arms to bind micelles and medications for a similar release outcome (Hou, 2022).

Beyond serving as direct medication delivery systems, polymer nanomicelles can also function as auxiliary materials. They may not act as direct delivery carriers but become a component of these carriers. For instance, in a micelle-gel system, micelles can be encapsulated in hydrogels. Complexes formed by medication-loaded polymer nanomicelles and *in-situ* gel polymerization are utilized for disease treatment and research. *In-situ* gels feature convenient administration and precise dosing. Compared to regular eye drops, they extend the residence time of medications in the eyes, reduce administration frequency, improve bioavailability, and achieve slow, long-term effects. These systems, liquid under storage conditions, transform into gels upon insertion into the eyes due to thermally responsive polymers’ phase transition properties (Bhowmik et al., 2011). Pluronics is a frequently used thermally responsive polymer composed of triblock copolymers. The copolymer exhibits amphiphilicity due to its hydrophilic polyethylene oxide (PEO) domain and hydrophobic polypropylene oxide (PPO) domain. The combination of Pluronics®F68 and Pluronics®F127 forms a hybrid polymeric micelle (MPM), an ideal nano-carrier system for insoluble medications (Sun et al., 2020). Additionally, Pluronics gelation varies with the micelle structure, influenced by the type, concentration, and temperature of Pluronics (Hamed, Al-Adhami, and Abu-Huwaij, 2019; Obaidat, Abu Kwiak, and Hamed, 2022). Pluronics®F127, renowned for its thermal gelation attributes and widespread commercial accessibility, is capable of creating thermoreversible hydrogels (TRGs) with a diminished critical gelation concentration (CGC) upon amalgamation with the innovative, thermally adaptive, and biocompatible terpolymer OEGMA30015-b-BuMA26-b-DEGMA13. This emergent methacrylate polymer showcases exceptional biocompatibility, and presents substantial promise as a candidate for injectable intraocular drug delivery formulations. (Constantinou et al., 2022).

Conducting polymer is an electroactive material capable of medication loading. Electrical stimulation can modify the medication delivery rate of conductive polymers (Abidian, Kim, and Martin, 2006; Luo and Cui, 2009) due to changes in the polymer backbone’s charge, polymer bulk volume, molecular permeability, and the polymer’s hydrophilic/hydrophobic balance (George et al., 2005). Despite the extensive reporting of these systems’ electrochemical polymerization, polymerization is restricted to the size of the conductive substrate upon which the film is polymerized. Recent studies show that chemical polymerization surpasses these limitations, as it does not require a conductive substrate, leads to the formation of insoluble polymer precipitates in solution, and allows rapid production of large quantities of conductive polymers, rendering it suitable for amplification (Ramanavičius, Kaušaitė, and Ramanavičienė, 2005). Conducting polymers such as polypyrrole (PPy), commonly prepared by electrochemical polymerization, can be utilized as electrically responsive medication delivery systems. Sodium dodecylbenzene sulfonate (SDBS) micelles loaded with the anionic, hydrophilic medication dexamethasone, and the nonionic, hydrophobic medication dexamethasone sodium phosphate are prepared. Chemical polymerization of PPy particles using these medication loaded SDBS micelles forms systems capable of regulating medication release in various scenarios, including chronic retinal diseases (Uppalapati et al., 2018). Table 1 provides a summary of eye-responsive drug delivery systems reported in recent literature (Jaiswal, Kumar, and Pathak, 2015; Chen et al., 2018; Devi et al., 2019; Wang et al., 2020; Hamed et al., 2022; Ch et al., 2023).

**TABLE 1:** Polymer nanomicelles based ophthalmic delivery systems in recent literature.

Delivery system	Polymers	Characteristics	Size (nm)	Entrapment efficiency	Model	References
pH-responsive polymer nanomicelles	Methoxy poly (ethyleneglycol)-poly (ε-caprolactone)-chitosan-TCA	pH responsive chitosan micelles;	50	2.4 μg/cm^2^	The HLECs	(Chen et al., 2018)
		pH induces effective release of antibacterial drugs;			L-929 fibroblast cells	
		Treatment of bacterial Keratitis				
	Pluronic®F127	polymeric micelles incorporated *in situ* ocular gel;	79.99	82.22% ± 1.91%	Goat cornea	(Jaiswal, Kumar, and Pathak, 2015)
		pH induces effective release of antibacterial drugs;				
		pH sensitive material carbopol934P;				
		Treatment of fungal Keratitis				
	Pluronic®F127	pH induces effective release of antibacterial drugs;	92	83.22 %± 2.39%	Albino rabbits, goat cornea	(Devi et al., 2019)
		pH sensitive material Carbopol940;				
		Treatment of allergic Conjunctivitis				
Temperature-responsive polymer nanomicelles	Chitosan-poly (lactide-co-glycolide)/poloxamer	Temperature sensitive mixture micelles; treating of bacterial cornea	127.0 ± 2.0	79.2%	Goat cornea	(Ch et al., 2023)
	mixed micelles				Balb/c mice	
	methoxyl polyethylene glycol-block-poly (benzyl glutamate) copolymer and CS-based thermo-responsive hydrogel	Designed CS-based thermosensitive hydrogel; used for optic nerve injury	150–500	1 mg/mL	Japanese white rabbits	(Wang et al., 2020)
	Pluronics^®^ F68	Thermal responsive *in situ* gel. Improving drug bioavailability	20.1–23.9	99.3%–106.3%	American white rabbits	(Hamed et al., 2022)
	Pluronics ®F127	extend the retention time of eye medication				
Electrochemical polymerization	PPy and SDBS micelles	Electric response triggers the release of loaded drugs	50	DexP (58.3% ± 2.50%), Dex (80.5% ± 1.19%)	ARPE-19 cell	(Uppalapati et al., 2018)

## 4 Application in eye diseases

**FIGURE 3:**
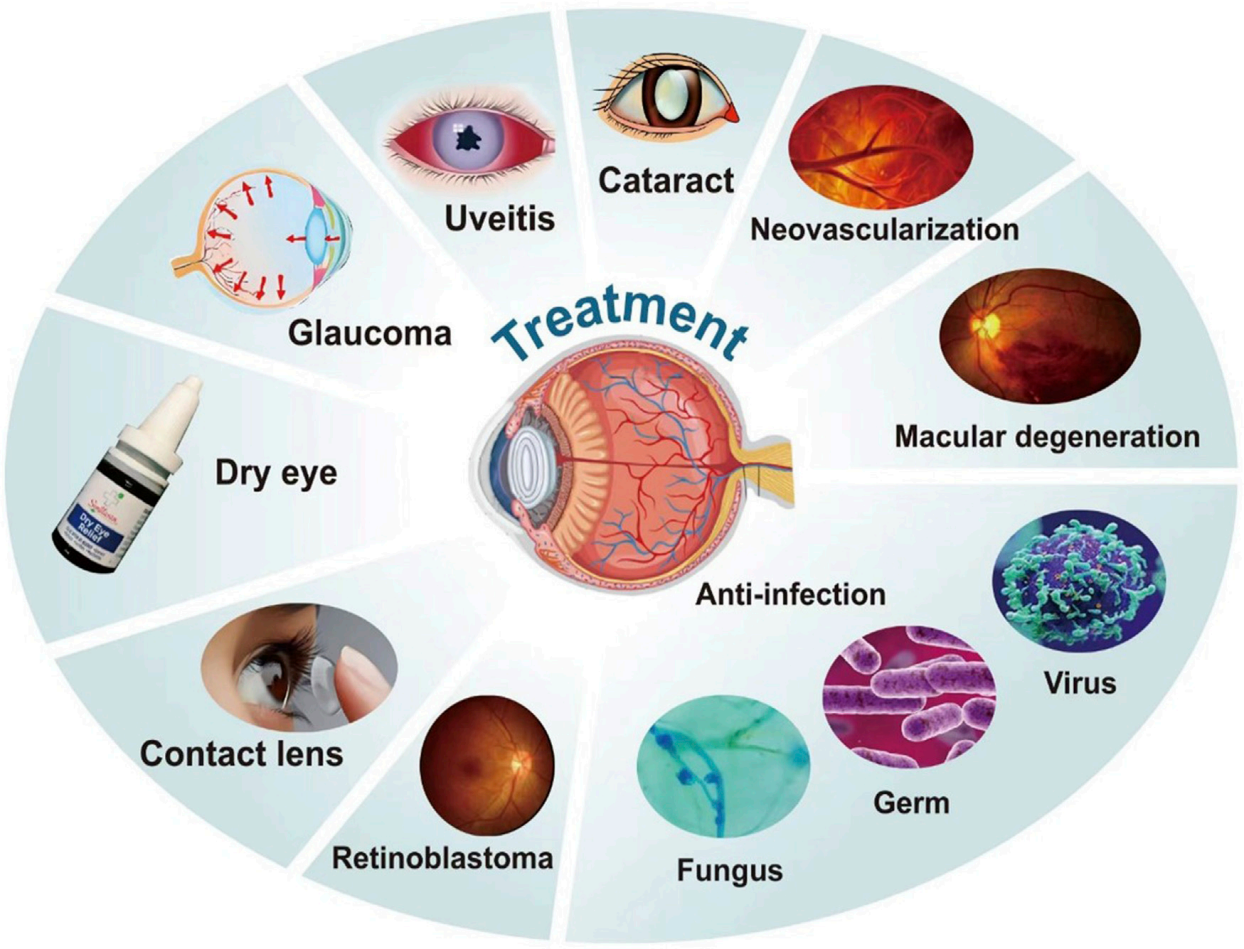
The treatment of eye diseases with polymer nanomicelles.

Polymer nanomicelles, a novel ocular medication delivery system, offer a multitude of benefits including increased solubility of insoluble medications, enhanced bioavailability of medications in ocular tissues, elongation of corneal residence time of medications, reduction in medication quantities, improved patient compliance, and minimized adverse medication reactions, marking a promising avenue in ocular medication delivery systems. However, certain limitations persist, such as suboptimal stability, limited medication loading, and significant ocular irritation. A majority of these systems remain in the experimental phase, with relatively few having made their way into clinical practice. Further exploration is needed to address these limitations, in terms of reducing adverse reactions of medication carriers, boosting medication loading, facilitating medication delivery to the posterior segment of the eye, and ensuring the safety and efficacy of the formulations. The research landscape of the diagnostic and therapeutic applications of polymer nanomicelles in ocular pathologies is summarized and evaluated (Figure 3).

Polymer nanomicelles carriers facilitate the delivery of large-molecule drugs, leveraging the process of endocytosis to discharge the encapsulated medication (Maysinger et al., 2007). It has been demonstrated that the copolymer, Poly (ethylene glycol)-Poly (lactic acid) (PEG-PLA), initially disassembles into fragments of PEG and PLA, with the remaining substances subsequently purged from the body (Cai et al., 2022). The degree to which a drug can penetrate cell membranes and its residence duration on the ocular surface constitute the two pivotal factors impacting the ultimate amount of drug that permeates cells. Owing to their inherent amphiphilic attributes, drug-loaded micelles are endocytosed, and under the impact of intracellular enzymes, the micelles disintegrate, thereby liberating the drug. The vast majority of studies primarily concentrate on the drug, thus leaving the integrity of the micelles during the absorption process under investigated. Current research pertaining to the distribution and elimination of ocular polymeric micelle drugs resorts to indirect or qualitative methods (Del Amo et al., 2017), indicating the existence of unresolved mysteries in this field that warrant further. in-depth examination. Current literature predominantly focuses on animal models or tissue experimental data. To further corroborate the safety, efficacy, and pharmacokinetics in human usage, future studies should emphasize strengthening relevant clinical trials.

### 4.1 Fungal infections

The phrase ‘ocular fungal infection’ embraces a spectrum of ocular tissue afflictions precipitated by a range of fungi, spanning superficial, deep, to conditionally pathogenic varieties. Fungal corneal infections hold the highest prevalence, succeeded by fungal blepharitis, endophthalmitis, dacryocystitis, and diseases of the orbit. Systemic antifungal therapeutics are not designated as the primary course of treatment for fungal keratitis, owing to the necessity of substantial doses to attain therapeutic concentrations within the targeted ocular tissues, which could potentially yield undesirable side effects (Fetih, 2016). The three main antifungal medications employed for fungal keratitis include polyenes (amphotericin B and nystatin), azoles (imidazole and triazole), and fluorocytosine (5-fluorocytosine) (Kaur, Rana, and Singh, 2008). Nonetheless, the application of these medications in the clinical management of fungal keratitis is restricted by their low corneal permeability, stability, and solubility (Li et al., 2018). Therefore, the development of polymer nanomicelles-based medication delivery systems can effectively address these limitations of conventional drug delivery methods.

Voriconazole, a broad-spectrum antifungal medication exhibiting activity against most fungi including *Candida* and Aspergillus, is employed in the treatment of fungal keratitis owing to its potent antifungal effect and excellent corneal permeability (Kumar and Sinha, 2017; Prajna et al., 2017). Despite its effectiveness, the poor water solubility of voriconazole has hindered the production of related eye drops on the market. Sun (Sun et al., 2022) pioneered a polymeric micelle composed of a phenylboronic acid-coupled chitosan-vitamin E copolymer (PBA-CSVE) and voriconazole. Chitosan, characterized by its low toxicity, eye tolerance, excellent bioadhesion, permeability, and prolonged medication retention on the eye surface, presents as an ideal choice for eye medication carrier materials. Vitamin E (VE), a potent antioxidant, is employed as the lipophilic end of polymer nanomicelles. Owing to its excellent lipid solubility, VE serves as a solvent for poorly soluble hydrophobic medications and provides a stabilizing effect on corneal cells. The most common method for achieving mucoadhesion involves forming electrostatic interactions between sialic acid residues on negatively charged corneal proteins and cationic polymers like chitosan. However, counter-ions in tears may thwart these electrostatic effects, leading to the washing away of these nanoparticles by tears. Phenylboronic acid (PBA), possessing a phenyl substituent and two hydroxyl groups attached to boron, can form complexes with diol groups on sialic acid at physiological pH (Prosperi-Porta et al., 2016; Cao et al., 2019). PBA molecules were utilized to modify the surface of nano-microcells to specifically target and bind sialic acid residues in the eye mucosa. The polymer nanomicelles were tested both *in vivo* and *in vitro* using the HCE-T cell line and New Zealand rabbits. The resultant PBA-CSVE loaded voriconazole polymeric micelles demonstrated a significant therapeutic effect, water solubility, biodegradability, low toxicity, and robust mucosal adhesion. The drug loading capacity of the micelle carrier is documented as 7.90% ± 0.02%. *In vitro* assessments using the HCE-T cell line revealed that the accumulated release rate of voriconazole amounted to 75% within the initial 0.5 h, with the drug almost completely discharged within a period of 2 h. It is beneficial for VRC to achieve an effective concentration of antifungal activity at the target site and reduce the development of drug resistance.

Mixed micelles represent a novel medication delivery system comprised of two or more distinct block copolymers or surfactants. This configuration offers superior thermodynamic and kinetic stability, and an enhanced capacity to encapsulate low water-soluble medications. Research on voriconazole-related ocular polymer nanomicelles is currently in full swing. It has been studied that voriconazole and two types of Pluronics (including PluronicP123 and PluronicF68) use Labrasol^®^ as simultaneous stabilizers to establish a mixed polymeric micelle delivery system. Herein, the weight ratio of Pluronics and voriconazole is 22.89:1, including 1:1 Pluronic®P123 and F68, and 2% w/v Labrasol^®^. Studies using New Zealand rabbits demonstrated effective eye mucosal adhesion and slow medication release (Fahmy et al., 2021). An *in-situ* gel system of polymer nanomicelles, prepared by loading itraconazole on Pluronic®F 127 and Pluronic®F 68, showed no irritation or corneal toxicity in goat cornea. It could overcome the disadvantages of commercial eye drops (Itral^®^) because of the addition of carbopol934P and the advantages of pH-responsive drug delivery (Jaiswal, Kumar, and Pathak, 2015). Key parameters associated with the polymer nanomicelles include: minimum micelle particle size (79.99 nm), maximum encapsulation efficiency (91.32% ± 1.73%), and an *in vitro* permeability rate over an 8-h period (90.28% ± 0.31%). The polymer nanomicelles maintain acceptable stability after being stored at ambient temperature for 3 months and exhibit higher and more persistent growth inhibition against fungi.

Phospholipid-based mixed micelles (MM) featuring egg phosphatidylcholine (EPC) as the primary component, in combination with various bile salts (sodium cholate (NaC), sodium deoxycholate (nano-DC), sodium taurocholate (NaTC)) or nonionic surfactants (Pluronic®F127, Pluronic®F68, Tween80, Labrasol®ALF, and d-a-tocopherol polyethylene glycol 1,000 succinate (TPGS)) were prepared to enhance the ophthalmic administration of the broad-spectrum antifungal medication, posaconazole (POS). Studies have shown that phospholipid-based mixed micelles significantly inhibit P-glycoprotein (P-gp)-mediated drug efflux (Osouli et al., 2023). The mean particle size of the polymer nanomicelles approximates 58 nm, demonstrating optimal stability for over a month, a gradual release pattern devoid of any noticeable initial burst, and an elevated level of *in vitro* antifungal activity.

Tolnaftate (TOL), a selective fungicide against Aspergillus, is a lipophilic medication with low water solubility and permeability. TOL polymeric pseudorotaxanes were designed and formulated by combining Tolnaftate and Pluronics. Both *in vivo* and *in vitro* experiments demonstrated that this type of drug-loaded polymer mixed micelle offers high bioavailability, low irritation, excellent eye histocompatibility, and significantly enhanced corneal flux (Aziz et al., 2022).

Solutol^®^ HS-15 (HS-15) is frequently employed to dissolve insoluble drugs, distinguished by its high stability, excellent biocompatibility, enhanced mucosal permeability, superior solubilization ability of hydrophobic drugs, and capacity to alter the pharmacokinetics of numerous medications (Hou et al., 2016). Studies indicate that HS-15 also exists in p-glycoprotein, influencing the cell membrane and opening the tight junctions between corneal epithelial cells. This enhances drug absorption through extracellular and paracellular mechanisms (Brayden et al., 2012), as well as the permeability of low molecular hydrophobic drugs and medium hydrophilic macromolecular drugs across epithelial cells, with low cytotoxicity (Shubber et al., 2015). Based on the animal experimental study of a binary mixed micelle of Solutol®HS-15 loaded with Sertaconazole nitrate, it is proven that its polymeric micelle model exhibits high physical stability, good biocompatibility, and enhanced corneal osmosis and eye delivery (Younes, Abdel-Halim, and Elassasy, 2018). The study of HS-15 polymer nanomicelles drug delivery system loaded with terbinafine hydrochloride showed that the polymer nanomicelles not only effectively penetrated the cornea but also demonstrated high drug loading and a small enough volume (Zhou et al., 2017). The drug encapsulation rate of the polymer nanomicelles nears 100%. The release of terbinafine from the micelles is contingent upon the pH; in a PBS solution with a pH of 5.0, around 93.2% ± 3.4% of the ensnared drug is liberated from the micelles within a 6-h period.

A novel organic-inorganic hybrid polymer was designed, comprising a polyethylene glycol-polypropylene glycol (PEG-PPG) copolymer modified by polyhedral oligomeric silsesquioxane (POSS) group, with amphotericin B (AMB) encapsulated in the polymer nanomicelles. POSS, a new generation of nanostructures with good biocompatibility, offers potential for prolonging the residence time of micelles on the eye surface. The micelle formed by the amphiphilic copolymer can load insoluble drugs and effectively improve the solubility of drugs. Using the mouse model of fungal keratitis, it was shown that POSS micelles loaded with AMB exhibited good safety and low irritation during treatment.

### 4.2 Bacterial infections

Bacteria are key perpetrators of ocular infections, with most infections occurring on the eye surface. However, intraocular infections, while less frequent, are potentially more detrimental, leading to irreversible visual impairment within hours to days. Eye infections primarily arise from Gram-positive (80%) and Gram-negative bacteria (20%), including common pathogens such as *Staphylococcus*, *Streptococcus*, *Pseudomonas aeruginosa*, and *Escherichia coli*. The widespread misuse of antibacterial medications has led to an alarming surge in drug resistance, necessitating vigilant monitoring in ophthalmology clinics. Untimely treatment of eye infections can result in severe complications. Broad-spectrum antibiotics are typically the mainstay of treatment for bacterial infections.

Erythromycin (ERY), a macrolide antibiotic, is widely employed to address bacterial eye infections, especially for those allergic to penicillin. ERY is predominantly available as an eye ointment as it has low water solubility, necessitating appropriate carriers for stable administration (Brisaert, Heylen, and Plaizier-Vercammen, 1996; Bhat, Dar, and Rather, 2008). Patients often exhibit poor compliance with ERY eye ointment due to temporary blurred vision and discomfort upon administration (Sipos et al., 2022). To overcome these issues, polymer nanomicelles loaded with ERY were developed with a micelle size of 87.14 nm and an encapsulation efficiency of 86.94%. These were dispersed in a Carbopol 934P gel matrix to prolong the drug release and permeation profile of ERY, thereby mitigating the ‘burst effect’. An innovative medication delivery system was subsequently formulated by integrating Soluplus^®^(SP), Kolliphor®HS-15 (KHS-15), Carbopol^®^934P, and ERY to prepare drug-loaded polymer nanomicelles. Combining the superior solubilization properties of SP with the permeability enhancement provided by KHS 15 offers a potent high-permeability ocular drug delivery solution, offering an innovative treatment modality for bacterial eye infections.

Bacterial keratitis is a rapidly evolving corneal ulceration that endangers vision, necessitating swift and efficient treatment. Sanjay (Ch et al., 2023) devised chitosan-poly (lactide-co-glycolide)/poloxamer mixed micelles functioning as mucoadhesive thermos responsive moxifloxacin eye drops to evaluate its therapeutic efficacy against bacterial keratitis. Human corneal epithelial (HCE) cells and goat corneas were used for *in vitro* and *in vivo* simulation experiments, respectively. The study findings revealed that the coalescence of poloxamer and polymer nanomicelles in a 1:10 ratio manifested superior physicochemical attributes, augmented mucosal adhesiveness, elevated corneal permeation, and demonstrated robust antibacterial potency, corroborated by both *in vitro* and *in vivo* examinations. Chitosan, apart from exhibiting effective mucosal adhesion, also displays biocompatible antibacterial and wound-healing characteristics (Itoo et al., 2022), providing pivotal insights for future research on related polymer nanomicelles.

Infectious keratitis is a stubborn disease precipitated by bacterial infection post-corneal trauma and is challenging to manage due to persistent infection and continuous inflammation typified by high concentrations of reactive oxygen species (ROS). To address this, poly (phenylborate-(3, 4-dihydropyrimidin-2 (1H)-ketone) co-(2-lactoaminoethyl methacrylate) (p (PBA-DHPM-r-LAMA)) sugar copolymeric micelles were developed for synergistic antibacterial and wound healing in bacterial keratitis. Levofloxacin (LEV) and chondroitin sulfate (CS) were co-encapsulated into antioxidant sugar polymer nanomicelles, with *in vitro* and *in vivo* simulation experiments carried out using human corneal epithelial cells and rat eyes. Under bacterial induction, the polymer micelles achieved an estimated 83% cumulative release over 48 h. The studies demonstrated that the drug-loaded polymer nanomicelles could achieve a trifecta of sterilization, ROS elimination, and wound healing (Zhang et al., 2022) paving the way for an effective antibacterial and wound-healing treatment strategy.

Ferulic acid demonstrates antibacterial efficacy against both Gram-negative and Gram-positive bacteria (Brannigan and Khutoryanskiy, 2017) and can be harnessed as an antibacterial agent in eye drops. Encapsulating ferulic acid within Pluronic^®^ micelles enhances its solubility, stability, and corneal permeability, and it also inhibits P-glycoprotein efflux in ocular tissues (Grimaudo et al., 2020).

### 4.3 Viral infections

Ocular infections caused by the varicella-zoster virus (VZV) predominantly manifest as keratitis (76.2%), uveitis (46.6%), and conjunctivitis (35.4%) (Yawn et al., 2013; Zhu and Zhu, 2014). Cytomegalovirus can precipitate retinitis, resulting in progressive visual loss and blindness in immunocompromised individuals (Scholz, Doerr, and Cinatl, 2003). Acyclovir, due to its low water solubility, could benefit from methods that enhance its solubility and ocular penetration. Soluplus, a biodegradable block copolymer, can serve as a matrix in solid solutions and impart *in-situ* gelation ability to aqueous dispersions, thereby extending persistence time on the ocular surface and controlling drug release (Alvarez-Rivera et al., 2016). Encapsulating acyclovir in Soluplus or Solutol polymer nanomicelles, as shown by animal model studies, increases its solubility, corneal permeability, and scleral permeability (Varela-Garcia, Concheiro, and Alvarez-Lorenzo, 2018).

### 4.4 Dry eye

Dry eye disease manifests through various symptoms, most commonly dryness and foreign body sensation in the eyes. Other symptoms may include burning, itching, light sensitivity, congestion, pain, blurred vision, fatigue, and sticky filamentous secretion. The primary aim of treating dry eye is to alleviate these symptoms, typically through non-medicated artificial tears, eye drops, and lubricants designed to maintain ocular surface moisture. In severe cases, immunosuppressive medications such as cyclosporine may be required.

Cyclosporine A (CsA), an immunomodulatory drug, is often employed in the treatment of intermediate and posterior eye diseases, including dry eye and uveitis. CsA, a hydrophobic molecule, presents challenges when incorporated into conventional local eye delivery systems. Cequa^®^, a 0.09% eye solution of CsA, has been approved by the US Food and Drug Administration (FDA) for dry eye treatment. Restasis^®^, the first CsA eye product approved by the FDA in 2003, has been associated with eye burning and pain (Schultz, 2014). Other significant drawbacks of Restasis^®^ include poor ocular tolerance, low bioavailability, and instability (Gupta and Chauhan, 2011; Karn et al., 2014; Schultz, 2014). To counter these limitations, recent studies have explored various cyclosporine polymer nanomicelles designed to enhance bioavailability, drug stability, corneal penetration, and ocular tolerance.

One such study by Martina et al. (Ghezzi et al., 2022) utilized Cyclosporine A, tocopherol polyethylene glycol 1,000 succinate (TPGS), and Solutol®HS-15 to create CsA polymer nanomicelles. The effectiveness of these micelles was demonstrated in animal studies. Micelles composed of TPGS have been evidenced to augment both the penetration and retention of drugs within the cornea and sclera, thereby functioning as a depot for drug delivery into these tissues. This continuous drug diffusion into deeper tissues enables a reduction in administration frequency, thereby bolstering patient adherence to treatment regimens. Furthermore, TPGS facilitates the release of vitamin E and vitamin E succinate, and its antioxidant activity may be beneficial in treating oxidation-mediated diseases.

In other studies, CsA polymeric micelles were prepared using cyclosporine A, octyl phenoxy poly (vinyloxy) ethanol (OPPEE, IGEPAL^®^ CA-630), and TPGS (Terreni et al., 2021). Furthermore, oxy polyethylene glycol-polylactide polymer (mPEG-PLA) micelles were employed as drug carriers for CsA solubilization and ocular delivery (Yu et al., 2018).

Myricetin (Myr), a natural flavonol compound, exhibits anti-inflammatory properties that are advantageous in treating eye degenerative and inflammatory diseases, including dry eye and chronic anterior uveitis. Encapsulating Myr into micelles in the form of self-assembled PVCL-PVA-PEG polymer facilitates the preparation of eye solutions. *In vivo* rabbit studies confirmed that these polymeric micelles are non-irritating and well-tolerated. PVCL-PVA-PEG micelles displayed superior corneal permeability *in vivo* compared to HS-15 micelles reported previously. These micelles could enhance the water solubility, stability, corneal permeability, and anti-inflammatory efficacy of Myr (Sun et al., 2019). PEG-DSPE/Solutol HS-15 mixed micelle and Curcumin were employed to prepare ocular polymeric micelle (Cur-MM-ISG). The experiment demonstrated that Cur-MM-ISG produced no apparent irritation in rabbit eyes post-application and exhibited prolonged residence time on the corneal surface (Sai et al., 2019).

Melatonin (N-acetyl-5-methoxytryptamine, Mel), an indoleamine hormone with diverse effects on ROS clearance, circadian rhythm maintenance, and immune response, can be used to treat dry eye. Melatonin-loaded PVCL-PVA-PEG polymeric micelles (Mel-Mic) have been shown to effectively combat oxidative stress induced by dry stress (DS) *in vivo* and hypertonic stress *in vitro* in mouse models. This formulation enhanced the reduction of ocular surface damage in mouse models and inhibited cell death in human corneal epithelial cells (HCCCs) (Saydam et al., 2017; Kim et al., 2019; Xu et al., 2022).

Tacrolimus (FK506), a hydrophobic macrolide antibiotic, inhibits the overactivation of immune response and can treat multiple ocular conditions, including dry eye. The physicochemical properties of tacrolimus-loaded mPEG-bPLGA micelles were assessed in rabbit corneas, demonstrating that these biocompatible micelles significantly enhanced the corneal penetration of tacrolimus while ensuring slow release to maintain an adequate dose. Consequently, mPEG-b-PLGA micelles could provide an effective ocular drug delivery system for hydrophobic medications (Zawadzka et al., 2012). In PBS fluid at pH values of either 7.4 or 5.0, a maximum of 80% of tacrolimus is discharged from the micelles within 48 h. The initial phase of tacrolimus release may be correlated with the dissociation of tacrolimus adhered to the surface. The *in vitro* release time of tacrolimus loaded with mPEG-b-PLGA is protracted, potentially reducing the administration frequency.

### 4.5 Cataract

Currently, the only established clinical treatment for cataracts involves the removal of the opaque lens followed by the implantation of an intraocular lens. Unfortunately, due to a lack of sophisticated medical infrastructure and specialized ophthalmologists in some developing regions, patients may not receive timely treatment. Consequently, alternative, cost-effective treatments with simpler management methods are in high demand for cataract prevention and treatment.

Nifedipine, a widely used, long-acting vasodilator traditionally administered to alleviate angina symptoms, has been suggested as a potential intervention in cataract management. It is postulated that calcium channel blockers like nifedipine may play a significant role in preventing oxidative cataract formation by limiting extracellular calcium influx. In a new type of eye drop formulation, nifedipine (NFP) is encapsulated within PLA-PEG micelles to prevent the onset and progression of early oxidative cataracts. NFP-loaded PLA-PEG micelles enhance biocompatibility and bioavailability while effectively inhibiting extracellular calcium ion flow, thereby bolstering anti-cataract capabilities (Xu, Qiu, et al., 2019).

Furthermore, Pluronic nano-micelles serve as a viable delivery platform for bromfenac sodium, a drug associated with various postoperative complications following cataract extraction. Studies on rabbits with corneal resection have validated the efficacy of micellar preparations comprising 5% w/v Pluronic F127% and 0.2% w/v hyaluronic acid. The encapsulated drug within these polymers exhibits enhanced ocular retention and penetration, thereby improving bioavailability (Patel et al., 2022).

### 4.6 Glaucoma

Glaucoma, characterized by optic nerve damage, stands as the second leading cause of blindness worldwide. Factors such as elevated intraocular pressure, optic nerve ischemia, and activation of oxidative stress-related pathways can contribute to glaucoma development. Local administration is typically the first line of treatment for glaucoma, despite significant limitations such as poor ocular bioavailability, which prevents reaching effective drug concentrations and may result in adverse reactions associated with the medication.

One promising approach involves encapsulating dorzolamide and indomethacin within poly (ε-caprolactone)-poly (N-vinylcaprolactam-co-N-vinylpyrrolidone) [PCL-g-P (NVCL-co-NVP)] graft copolymer to form polymeric micelles (PM) (Ozturk et al., 2022). *In vitro* biological tests revealed that dorzolamide increased micelle size, while indomethacin exhibited the opposite effect. These micelles displayed excellent blood and cell compatibility. Further experimentation in rabbits demonstrated that the designed micelles effectively reduced intraocular pressure.

A glaucoma model in rabbits was constructed using a methoxy-poly (ethylene glycol)-b-poly (ε-caprolactone) (mPEG-PCL) diblock copolymer loaded with methazolamide to form a polymer nanomicelles. The resulting micelle model showed high encapsulation efficiency, biocompatibility, and a substantial anti-glaucoma effect (Elmowafy et al., 2019). Additionally, Rebaudioside A/TPGS mixed nano-micelles were employed as nano-carriers for intraocular administration of nimodipine. The nimodipine polymeric micelle eye solution was investigated for its therapeutic potential, enhancing nimodipine’s prospects for use in glaucoma treatment in local eye preparations (Li, Fang, et al., 2021).

### 4.7 Neovascularization

Corneal neovascularization (CNV), a sequela of anterior section inflammation, can lead to compromised vision and even complete loss of sight. The pathogenesis of CNV involves an increased expression of angiogenic factors, including basic fibroblast growth factor (bFGF) and vascular endothelial growth factor (VEGF) (Gupta and Illingworth, 2011). Anti-inflammatory agents, particularly steroids such as prednisolone and dexamethasone, have served as the cornerstone of CNV therapy for years due to their robust capacity to hinder the activation, migration, and recruitment of vascular endothelial cells and inflammatory cells, including T-cell and macrophages (Li, Liu, Yu, et al., 2021).

A preparation consisting of Diclofenac (DIC) and an anti-VEGF antibody (Avastin^®^; Ava) was found to effectively suppress CNV due to their combined effect. They were encapsulated within heat-sensitive hydrogels (poly (DL-lactide)-poly (ethylene glycol)-poly (DL-lactide); PDLLA-PEG-PDLLA) and administered through a single subconjunctival injection using the rabbit CNV model. The resultant decrease in CNV was potentially associated with reduced inflammatory cell infiltration and VEGF expression (Shi et al., 2022).

Amphiphilic MPEG-PCL micelles were utilized to create acitinib-loaded micelles with excellent uniform dispersion. Cross-sectional comparisons of drug loading efficiencies among diverse molecular segments unequivocally revealed superior loading efficacy when the lengths of the hydrophilic and hydrophobic chains within the amphiphilic block polymer bore similarity. Furthermore, these drug-laden micelles demonstrated remarkable biocompatibility, thereby establishing the requisite conditions conducive to ocular anti-angiogenesis. Through the creation of an eye surface angiogenesis model, the potential application of this medication within the eyes was verified, offering a novel alternative for ocular therapeutics (Shi et al., 2019).

Genistein, a hydrophobic drug extensively used in the treatment of eye angiogenesis, was packed into an MPEG-b-PAE-g-HA carrier. Cell viability tests indicated that genistein micelles exhibited no significant cytotoxicity towards human corneal epithelial cells. A series of *in vitro* experiments confirmed the drug’s sustained release and corneal penetration capabilities, while vascular inhibition tests demonstrated the micelles’ significant suppression of human umbilical vein endothelial cell angiogenesis (Li, Chen, et al., 2018).

Imatinib, a drug known for inhibiting endothelial cell germination and promoting cell tube rupture, was encapsulated within micelles created by hyaluronic acid (HA) derivatives, containing ethylenediamine (EDA), hexadecyl (C16), polyethylene glycol (PEG), and/or L-carnitine (CRN). The resultant micelles-termed HA-EDA-C16, HA-EDA-C16-PEG, and HA-EDA-C16-CRN micelles-facilitated the loading of imatinib, thereby maintaining cell viability. Studies demonstrated that these polymeric micelles could interact with the corneal barrier, promoting transcorneal permeation of imatinib through non-invasive administration (Bongiovì et al., 2018).

Carbotinib, through the inhibition of the VEGF signaling pathway, has shown promise as a potential treatment for CNV. For topical application, the lipophilic carbotinib might fail to reach the stromal layer - where CNV originates - due to the barrier posed by the hydrophilic tear film and hydrophobic corneal epithelial cells. Systemic administration or intraocular injection of carbotinib can, however, lead to systemic toxicity or ocular complications. This necessitates the urgent need for biocompatible and effective drug carriers for ocular delivery of carbotinib.

A cationic amphiphilic peptide, Lys-(NH2)-Phe, was successfully synthesized via NCA-ROP polymerization, serving as a nano-carrier to encapsulate the hydrophobic carbotinib. The cationic Cabo-NP was capable of penetrating the hydrophilic tear film and adhering to the corneal mucosal surface, thereby ensuring longer retention of carbotinib, enhancing bioavailability, and exhibiting no cytotoxicity or eye surface irritation. The inhibitory effect of Cabo-NP on angiogenesis was affirmed by VEGF-induced cell migration and tube formation assays. In a mouse model simulating alkali burn, Cabo-NPs reduced inflammatory infiltration, fibrosis, and angiogenic factors, thus enhancing the therapeutic effect on CNV (Han et al., 2020).

### 4.8 Uveitis

Uveitis, an intraocular inflammatory condition, leads to a range of visual impairments. Dexamethasone is the primary therapeutic agent used to alleviate uveitis symptoms. The integration of dexamethasone into PCL-PEG-PCL micelles enhances the sustained release and significantly improves corneal penetration compared to existing DEX eye drops (Alami-Milani et al., 2018). The PLA-PCL-PEG-PCL-PLA copolymer was synthesized, and dexamethasone-loaded polymer nanomicelles were generated using the o/w emulsion solvent evaporation technique. *In vitro* experiments demonstrated that these micelles significantly improved corneal permeability (Alami-Milani et al., 2020).

Polymer nanomicelles of poly (ethylene glycol)-block-poly (e-caprolactone) (PEG-b-PCL) and poly (ethylene glycol)-block-lactic acid (PEG-b-PLA) loaded with triamcinolone acetonide (TA) were created as potential therapeutics for ocular inflammation. Results from the rabbit model affirmed the potential of PEG-b-PLA micelles to augment the anti-inflammatory efficacy of triamcinolone acetonide (Safwat et al., 2020). PEG-b-PLA micelles suspended in a chitosan hydrogel are able to sustain drug release, with only 42.8 %± 1.6% of the drug released within a one-week interval.

### 4.9 Macular degeneration

Lutein, due to its free radical scavenging properties, plays a critical role in mitigating age-related macular degeneration, cataract complications, and diabetes-mediated retinopathy. Retinal conditions such as neovascular age-related macular degeneration and diabetic macular edema significantly contribute to global visual impairment. The traditional intravitreal injection of anti-vascular endothelial growth factor (anti-VEGF) into the posterior segment of the eye for retinal disorders is invasive and often triggers related complications.

The copolymer EPC (nEPCs), composed of poly (ethylene glycol) (PEG), poly (propylene glycol) (PPG), and poly (caprolactone) (PCL) segments, was engineered to encapsulate aflibercept, forming polymer nanomicelles. These micelles can reach therapeutic concentrations in the posterior segment of mouse eyes. The inherent anti-angiogenic properties of nEPCs may augment the anti-angiogenic effects of aflibercept (Zhao et al., 2022).

Statins are thought to be beneficial for age-related macular degeneration and immune and inflammatory disorders impacting the posterior segment of the eye. However, the available data pertains to oral administration. Although their ophthalmic administration might be advantageous, the safety and effectiveness of directly administering statins to the eye remain uncertain. Polymer nanomicelles based on TPGS or TPGS/poloxamer 407 enhance the solubility and stability of simvastatin, promoting drug delivery to the posterior segment of the eye through scleral penetration. In isolated pig models, TPGS micelles demonstrated superior efficiency in delivering simvastatin through the conjunctiva or sclera (Pescina et al., 2021). As for drug stability, the quantity of simvastatin entrapped within the polymer micelles diminishes gradually over time, particularly at elevated storage temperatures. In fact, after 1 month at 4 °C, roughly 80% of the original drug was detected, while this value precipitously plummeted to 30% when the micelles were stored at 25 °C.

### 4.10 Retinoblastoma

Celatrol, a Chinese herbal medicine, has inhibitory effect on the growth activity of Retinoblastoma. Celastrol nanomicelles (CNMs) inhibit the growth of retinoblastoma by inducing apoptosis of human Retinoblastoma SO-Rb 50 cells (Li et al., 2012). In addition, CNMs inhibit hypoxia induced proliferation, migration and invasion of human umbilical vascular endothelial cells. CNM inhibits the growth of Retinoblastoma in xenotransplantation mouse models by inhibiting tumor angiogenesis, which may be related to the inhibition in the HIF-Ia/VEGF pathway (Zhang et al., 2022).

### 4.11 Contact lens

Polymer nanomicelles, when incorporated into hydrogels and contact lenses, can enhance medication delivery. Mun and others (Mun et al., 2019) conceived micellar-embedded contact lenses to facilitate sustained medication release. They prepared pHEMA contact lenses via photopolymerization, introducing C-HA micelles loaded with cyclosporin into the polymerization mixture. *In vitro* analyses demonstrated that medications continue to be released from micelle-embedded lenses over a period exceeding 10 days. Further *in vitro* and *in vivo* studies affirmed the therapeutic efficacy of these specially designed contact lenses in treating dry eye syndrome.

Lu and team (Lu et al., 2013) designed a rod-shaped micelle lens to extend the delivery of dexamethasone acetate. Meanwhile, Xu and others (Xu et al., 2019) explored the concurrent delivery of timolol and latanoprost medications from micelle-embedded contact lenses. They mixed medication-loaded mPEG-PLA (micelle) into the HEMA monomer, obtaining medication-loaded micelle contact lenses through free radical polymerization. Incorporating polymer nanomicelles medications into contact lenses remains a focal area in the research for treating eye-related diseases.

## 5 Challenges with ocular polymer nanomicelles

Polymer nanomicelles delivery systems grapple with a range of challenges including inadequate stability, limited drug loading capacity, irritant excipients, and incomplete drug discharge. Nevertheless, it is anticipated that continued research efforts will alleviate these issues. With the expanding study on nanomedicines, the safety of polymeric micelle formulations has garnered increasing attention. A significant drawback of micelle systems necessitates the assessment of potential toxicity in long-term ocular surface application. Consequently, the development of a biocompatible and safer micelle drug delivery system is posited as a prospective research objective (Dvořáková et al., 2017). This article provides a succinct summary of the relevant issues regarding several ocular polymer micelles.

Research indicates that PEG-b-PLA micelles may exhibit potential toxicity and instigate neuroendocrine disruption (De Campos et al., 2003). Certain polymer nanomicelles incorporating polyethylene glycol not only showcase excellent biocompatibility and solubility but also evince good corneal penetration and sustained-release characteristics. Their limitation, however, lies in their inability to access deeper regions (such as the retina), necessitating injection administration and thereby limiting the usage of topical application. The majority of current research on polymeric micelles involves the establishment of animal and *in vitro* models, with interspecies variances in animal experimental data obstructing the clinical application research of these drugs. Two polymers, Soluplus and Pluronic F68, have been selected for micelle preparation. Soluplus is a graft copolymer of polyvinyl caprolactam-polyvinyl acetate-polyethylene glycol (PCL-PVAc-PEG). The penetration rate of polymeric micelle drugs is cornea-dependent, with drugs infiltrating more rapidly through rabbit cornea and sclera than through pig or cow cornea (Alambiaga-Caravaca et al., 2020). In practical application, the presence of numerous interfering molecules on the ocular surface can impact the sensitivity of gel stimulation-response, enhancing the unpredictability of the final gel formation (Cooke et al., 2018). Additional concerns encompass the transparency of the formed gel, regulatory complications concerning constituents, the non-degradability of the gel, and toxicity correlated with dosage. Literature reports propose that the gel barrier can influence drug release, resulting in reduced drug concentrations in the initial phase (Wu et al., 2013). Various strategies for drug-loading contact lenses strive to achieve drug loading, sustained release, and maximal extension of effective drug concentration duration on the ocular surface without compromising the fundamental properties of contact lenses. Translucency, oxygen permeability, mechanical properties, and ionic permeability substantially impact patient comfort and compliance. Therefore, these elements cannot be overlooked to enhance the clinical transition potential of drug-loaded contact lenses (Xu et al., 2018). In studies examining the ocular application of drug-loaded polymeric micelles, the occurrence of focal yellow vitreous and lens discoloration has been observed in some animals, becoming a considerable hurdle for clinical application (Tsujinaka et al., 2020).

## 6 Conclusion and prospects

Over the past few decades, the employment of polymer nanomicelles in ophthalmology has presented an effective method for medication delivery. They can significantly enhance the solubility of insoluble medications, tackling the issues associated with insoluble medications in ophthalmic diseases. Despite their potential, the clinical application of polymeric micelles faces significant challenges, with only a handful of polymeric micelle products currently in clinical use.

The unique anatomical structure of the eye and the existence of various barriers necessitate high biocompatibility and stability, further complicating clinical applications. However, compared to conventional ocular medication delivery preparations, these polymer nanomicelles can effectively surmount some eye barriers through local administration. They also improve medication loading, release capacity, and stability while utilizing related sensitive targets for early disease diagnosis.

Predominantly, medication research foundations lie within cell and animal model investigations. Future translation into clinical practice necessitates robust reinforcement of medication clinical trials, complemented by in-depth exploration into the safety profiles and pharmacokinetic parameters of polymeric micelles. An additional point of consideration involves potential irritation or toxicity engendered by the amphiphilic polymers themselves. Typically, research incorporates *in vitro* evaluations of isolated conjunctival or corneal cells, or vascularized chorioallantoic membranes of fertilized eggs. In a long-term perspective, the tolerability in human subjects after repeated application, particularly concerning polymers designated for ocular applications not yet sanctioned by regulatory bodies, requires affirmative confirmation.

The clinical trial of nano-medications is dependent on the implementation and validation of good manufacturing practices (GMP) to ensure the quality of nano-products. Yet, the challenge lies in the repeatability and scalability of the nano-film production method, making GMP compliance a struggle. Moreover, the regulatory landscape for nano-medications remains in its infancy, presenting a primary obstacle to the clinical application of polymer nanomicelles. As such, improvements may be necessary in the areas of manufacturing process regulation and process control, quality characterization, medication quality, product spectrum, and stability.

## References

[B1] Abidian M. R. Kim D. H. Martin D. (2006). Inside front cover: Conducting-polymer nanotubes for controlled drug release (adv. Mater. 4/2006). Adv. Mat. 18, 405–409. 10.1002/adma.200501726 PMC3088882 21552389

[B2] Addo R. T. Yeboah K. G. Siwale R. C. Siddig A. Jones A. Ubale R. V. (2015). Formulation and characterization of atropine sulfate in albumin–chitosan microparticles for *in vivo* ocular drug delivery. J. Pharm. Sci. 104, 1677–1690. 10.1002/jps.24380 25652269

[B3] Alambiaga-Caravaca A. M. Calatayud-Pascual M. A. Rodilla V. Concheiro A. López-Castellano A. Alvarez-Lorenzo C. (2020). Micelles of progesterone for topical eye administration: Interspecies and intertissues differences in *ex vivo* ocular permeability. Pharmaceutics 12 (8), 702. 10.3390/pharmaceutics12080702 32722548 PMC7464168

[B4] Alami-Milani M. Zakeri-Milani P. Valizadeh H. Fathi M. Salatin S. Salehi R. (2020). PLA-PCL-PEG-PCL-PLA based micelles for improving the ocular permeability of dexamethasone: Development, characterization, and *in vitro* evaluation. Pharm. Dev. Technol. 25 (6), 704–719. 10.1080/10837450.2020.1733606 32098567

[B5] Alami-Milani M. Zakeri-Milani P. Valizadeh H. Salehi R. Jelvehgari M. (2018). Preparation and evaluation of PCL-PEG-PCL micelles as potential nanocarriers for ocular delivery of dexamethasone. Iran. J. Basic. Med. Sci. 21 (2), 153–164. 10.22038/ijbms.2017.26590.6513 29456812 PMC5811754

[B6] Alvarez-Rivera F. Fernández-Villanueva D. Concheiro A. Alvarez-Lorenzo C. (2016). α-Lipoic acid in Soluplus ® polymeric nanomicelles for ocular treatment of diabetes-associated corneal diseases. J. Pharm. Sci. 105 (9), 2855–2863. 10.1016/j.xphs.2016.03.006 27103010

[B7] Anselmo A. C. Mitragotri S. (2016). Nanoparticles in the clinic. Bioeng. Transl. Med. 1 (1), 10–29. 10.1002/btm2.10003 29313004 PMC5689513

[B8] Anselmo A. C. Mitragotri S. (2019). Nanoparticles in the clinic: An update. Bioeng. Transl. Med. 4 (3), e10143. 10.1002/btm2.10143 31572799 PMC6764803

[B9] Ashley J. D. Quinlan C. J. Schroeder V. A. Suckow M. A. Pizzuti V. J. Kiziltepe T. (2016). Dual carfilzomib and doxorubicin-loaded liposomal nanoparticles for synergistic efficacy in multiple myeloma. Mol. Cancer. Ther. 15 (7), 1452–1459. 10.1158/1535-7163.Mct-15-0867 27196779

[B10] Aziz D. Mohamed S. Tayel S. Makhlouf A. (2022). Implementing polymeric pseudorotaxanes for boosting corneal permeability and antiaspergillus activity of tolnaftate: Formulation development, statistical optimization, *ex vivo* permeation and *in vivo* assessment. Drug. Deliv. 29 (1), 2162–2176. 10.1080/10717544.2022.2094499 35815689 PMC9278446

[B11] Bhat P. A. Dar A. A. Rather G. M. (2008). Solubilization capabilities of some cationic, anionic, and nonionic surfactants toward the poorly water-soluble antibiotic drug erythromycin. J. Chem. Eng. Data. 53 (6), 1271–1277. 10.1021/je700659g

[B12] Bhowmik M. Das S. Chattopadhyay D. Ghosh L. K. (2011). Study of thermo-sensitive *in-situ* gels for ocular delivery. Sci. Pharm. 79 (2), 351–358. 10.3797/scipharm.1010-04 21773071 PMC3134854

[B13] Bongiovì F. Fiorica C. Palumbo F. S. Prima G. D. Giammona G. Pitarresi G. (2018). Imatinib-Loaded micelles of hyaluronic acid derivatives for potential treatment of neovascular ocular diseases. Mol. Pharm. 15 (11), 5031–5045. 10.1021/acs.molpharmaceut.8b00620 30248267

[B14] Brannigan R. P. Khutoryanskiy V. V. (2017). Synthesis and evaluation of mucoadhesive acryloyl-quaternized PDMAEMA nanogels for ocular drug delivery. Colloids. Surf. B. Biointerfaces. 155, 538–543. 10.1016/j.colsurfb.2017.04.050 28494432

[B15] Brayden D. J. Bzik V. A. Lewis A. L. Illum L. (2012). CriticalSorb™ promotes permeation of flux markers across isolated rat intestinal mucosae and Caco-2 monolayers. Pharm. Res. 29 (9), 2543–2554. 10.1007/s11095-012-0785-6 22638869

[B16] Brisaert M. Heylen M. Plaizier-Vercammen J. (1996). Investigation on the chemical stability of erythromycin in solutions using an optimization system. Pharm. World. Sci. 18 (5), 182–186. 10.1007/bf00820730 8933579

[B17] Buschmann M. D. Carrasco M. J. Alishetty S. Paige M. Alameh M. G. Weissman D. (2021). Nanomaterial delivery systems for mRNA vaccines. Vaccines (Basel) 9 (1), 65. 10.3390/vaccines9010065 33478109 PMC7836001

[B18] Cai Y. Qi J. Lu Y. He H. Wu W. (2022). The *in vivo* fate of polymeric micelles. Adv. Drug. Deliv. Rev. 188, 114463. 10.1016/j.addr.2022.114463 35905947

[B19] Cao J. Gao X. Cheng M. Niu X. Li X. Zhang Y. (2019). Reversible shielding between dual ligands for enhanced tumor accumulation of ZnPc-loaded micelles. Nano. Lett. 19 (3), 1665–1674. 10.1021/acs.nanolett.8b04645 30801190

[B20] Caracciolo P. C. Diaz-Rodriguez P. Ardao I. Moreira D. Montini-Ballarin F. Abraham G. A. (2021). Evaluation of human umbilical vein endothelial cells growth onto heparin-modified electrospun vascular grafts. Int. J. Biol. Macromol. 179, 567–575. 10.1016/j.ijbiomac.2021.03.008 33675835

[B21] Ch S. Padaga S. G. Ghosh B. Roy S. Biswas S. (2023). Chitosan-poly(lactide-co-glycolide)/poloxamer mixed micelles as a mucoadhesive thermo-responsive moxifloxacin eye drop to improve treatment efficacy in bacterial keratitis. Carbohydr. Polym. 312, 120822. 10.1016/j.carbpol.2023.120822 37059521

[B22] Chen H. Ye Z. Sun L. Li X. Shi S. Hu J. (2018). Synthesis of chitosan-based micelles for pH responsive drug release and antibacterial application. Carbohydr. Polym. 189, 65–71. 10.1016/j.carbpol.2018.02.022 29580427

[B23] Constantinou A. P. Nele V. Doutch J. J. Correia J. S. Moiseev R. V. Cihova M. (2022). Investigation of the thermogelation of a promising biocompatible ABC triblock terpolymer and its comparison with pluronic F127. Macromolecules 55 (5), 1783–1799. 10.1021/acs.macromol.1c02123 35431333 PMC9007541

[B24] Cooke M. E. Jones S. W. Horst B. T. Moiemen N. Snow M. Chouhan G. (2018). Structuring of hydrogels across multiple length scales for biomedical applications. Adv. Mat. 30 (14), e1705013. 10.1002/adma.201705013 29430770

[B25] Craig M. Altskär A. Nordstierna L. Holmberg K. (2016). Bacteria-triggered degradation of nanofilm shells for release of antimicrobial agents. J. Mat. Chem. B 4 (4), 672–682. 10.1039/c5tb01274k 32262949

[B26] De Campos A. M. Sánchez A. Gref R. Calvo P. Alonso M. J. (2003). The effect of a PEG versus a chitosan coating on the interaction of drug colloidal carriers with the ocular mucosa. Eur. J. Pharm. Sci. 20 (1), 73–81. 10.1016/s0928-0987(03)00178-7 13678795

[B27] Del Amo E. M. Rimpelä A. K. Heikkinen E. Kari O. K. Ramsay E. Lajunen T. (2017). Pharmacokinetic aspects of retinal drug delivery. Prog. Retin. Eye. Res. 57, 134–185. 10.1016/j.preteyeres.2016.12.001 28028001

[B28] Devi S. Saini V. Kumar M. Bhatt S. Gupta S. Deep A. (2019). A novel approach of drug localization through development of polymeric micellar system containing azelastine HCl for ocular delivery. Pharm. Nanotechnol. 7 (4), 314–327. 10.2174/2211738507666190726162000 31362666 PMC7040519

[B29] Dvořáková M. Rollerová E. Scsuková S. Bujňáková Mlynarčíková A. Laubertová L. Žitňanová I. (2017). Effect of neonatal exposure to poly(ethylene glycol)-block-Poly(lactic acid) nanoparticles on oxidative state in infantile and adult female Rats. Oxid. Med. Cell. Longev. 2017, 1. 8. 10.1155/2017/7430435 PMC5610884 29081892

[B30] Elmowafy E. Gad H. Biondo F. Casettari L. Soliman M. E. (2019). Exploring optimized methoxy poly(ethylene glycol)-block-poly(ε-caprolactone) crystalline cored micelles in anti-glaucoma pharmacotherapy. Int. J. Pharm. 566, 573–584. 10.1016/j.ijpharm.2019.06.011 31176850

[B31] Enriquez G. G. Rizvi S. A. D'Souza M. J. Do D. P. (2013). Formulation and evaluation of drug-loaded targeted magnetic microspheres for cancer therapy. Int. J. Nanomedicine. 8, 1393–1402. 10.2147/ijn.S43479 23630421 PMC3626373

[B32] Fahmy A. M. Hassan M. El-Setouhy D. A. Tayel S. A. Al-Mahallawi A. M. (2021). Voriconazole ternary micellar systems for the treatment of ocular mycosis: Statistical optimization and *in vivo* evaluation. J. Pharm. Sci. 110 (5), 2130–2138. 10.1016/j.xphs.2020.12.013 33346033

[B33] Fetih G. (2016). Fluconazole-loaded niosomal gels as a topical ocular drug delivery system for corneal fungal infections. J. Drug. Deliv. Sci. Tec. 35, 8–15. 10.1016/j.jddst.2016.06.002

[B34] Franca J. R. Foureaux G. Fuscaldi L. L. Ribeiro T. G. Rodrigues L. B. Bravo R. (2014). Bimatoprost-loaded ocular inserts as sustained release drug delivery systems for glaucoma treatment: *In vitro* and *in vivo* evaluation. PLoS. One. 9 (4), e95461. 10.1371/journal.pone.0095461 24788066 PMC4005758

[B35] Galaev I. Y. Mattiasson B. (1999). Smart' polymers and what they could do in biotechnology and medicine. Trends. Biotechnol. 17 (8), 335–340. 10.1016/s0167-7799(99)01345-1 10407406

[B36] George P. M. Lyckman A. W. LaVan D. A. Hegde A. Leung Y. Avasare R. (2005). Fabrication and biocompatibility of polypyrrole implants suitable for neural prosthetics. Biomaterials 26 (17), 3511–3519. 10.1016/j.biomaterials.2004.09.037 15621241

[B37] Germain M. Caputo F. Metcalfe S. Tosi G. Spring K. Åslund A. (2020). Delivering the power of nanomedicine to patients today. J. Control. Release. 326, 164–171. 10.1016/j.jconrel.2020.07.007 32681950 PMC7362824

[B38] Ghezzi M. Ferraboschi I. Delledonne A. Pescina S. Padula C. Santi P. (2022). Cyclosporine-loaded micelles for ocular delivery: Investigating the penetration mechanisms. J. Control. Release. 349, 744–755. 10.1016/j.jconrel.2022.07.019 35901859

[B39] Giunchedi P. Chetoni P. Conte U. Saettone M. F. (2000). Albumin microspheres for ocular delivery of piroxicam. Pharm. Pharmacol. Commun. 6 (4), 149–153. 10.1211/146080800128735809

[B40] Grimaudo M. A. Amato G. Carbone C. Diaz-Rodriguez P. Musumeci T. Concheiro A. (2020). Micelle-nanogel platform for ferulic acid ocular delivery. Int. J. Pharm. 576, 118986. 10.1016/j.ijpharm.2019.118986 31870956

[B41] Grimaudo M. A. Pescina S. Padula C. Santi P. Concheiro A. Alvarez-Lorenzo C. (2019). Topical application of polymeric nanomicelles in ophthalmology: A review on research efforts for the noninvasive delivery of ocular therapeutics. Expert. Opin. Drug. Deliv. 16 (4), 397–413. 10.1080/17425247.2019.1597848 30889977

[B42] Gupta C. Chauhan A. (2011). Ophthalmic delivery of cyclosporine A by punctal plugs. J. Control. Release. 150 (1), 70–76. 10.1016/j.jconrel.2010.11.009 21074586

[B43] Gupta D. Illingworth C. (2011). Treatments for corneal neovascularization: A review. Cornea 30 (8), 927–938. 10.1097/ICO.0b013e318201405a 21389854

[B44] Gupta P. Vermani K. Garg S. (2002). Hydrogels: From controlled release to pH-responsive drug delivery. Drug. Discov. Today. 7 (10), 569–579. 10.1016/s1359-6446(02)02255-9 12047857

[B45] Hamed R. Al-Adhami Y. Abu-Huwaij R. (2019). Concentration of a microemulsion influences the mechanical properties of ibuprofen *in situ* microgels. Int. J. Pharm. 570, 118684. 10.1016/j.ijpharm.2019.118684 31505215

[B46] Hamed R. Kwiak A. D. A. Al-Adhami Y. Hammad A. M. Obaidat R. Abusara O. (2022). Microemulsions as lipid nanosystems loaded into thermoresponsive *in situ* microgels for local ocular delivery of prednisolone. Pharmaceutics 14, 1975. 10.3390/pharmaceutics14091975 36145726 PMC9505494

[B47] Han H. Yin Q. Tang X. Yu X. Gao Q. Tang Y. (2020). Development of mucoadhesive cationic polypeptide micelles for sustained cabozantinib release and inhibition of corneal neovascularization. J. Mat. Chem. B 8 (23), 5143–5154. 10.1039/d0tb00874e 32420566

[B48] Hizal F. Zhuk I. Sukhishvili S. Busscher H. J. Mei H. C. Choi C. H. (2015). Impact of 3D hierarchical nanostructures on the antibacterial efficacy of a bacteria-triggered self-defensive antibiotic coating. Acs. Appl. Mat. Interfaces. 7 (36), 20304–20313. 10.1021/acsami.5b05947 26305913

[B49] Hoffman A. S. Stayton P. S. Chen G. Chen J. Cheung C. Chilkoti A. (2000). Really smart bioconjugates of smart polymers and receptor proteins. J. Biomed. Mat. Res. 52, 577–586. 10.1002/1097-4636(20001215)52:4<577::AID-JBM1>3.0.CO;2-5 11033539

[B50] Hou J. Sun E. Sun C. Wang J. Yang L. Jia X. B. (2016). Improved oral bioavailability and anticancer efficacy on breast cancer of paclitaxel via Novel Soluplus(®)-Solutol(®) HS15 binary mixed micelles system. Int. J. Pharm. 512 (1), 186–193. 10.1016/j.ijpharm.2016.08.045 27567930

[B51] Hou L. (2022). Ressarch progress on PH-sensitive polymeric micelle for drug delivery. Guangzhou Chem. Ind. 50 (09), 14–16+22.

[B52] Hwang D. Ramsey J. D. Kabanov A. V. (2020). Polymeric micelles for the delivery of poorly soluble drugs: From nanoformulation to clinical approval. Adv. Drug. Deliv. Rev. 156, 80–118. 10.1016/j.addr.2020.09.009 32980449 PMC8173698

[B53] Itoo A. M. Paul M. Ghosh B. Biswas S. (2022). Oxaliplatin delivery via chitosan/vitamin E conjugate micelles for improved efficacy and MDR-reversal in breast cancer. Carbohydr. Polym. 282, 119108. 10.1016/j.carbpol.2022.119108 35123744

[B54] Jaiswal M. Kumar M. Pathak K. (2015). Zero order delivery of itraconazole via polymeric micelles incorporated *in situ* ocular gel for the management of fungal keratitis. Colloids. Surf. B. Biointerfaces. 130, 23–30. 10.1016/j.colsurfb.2015.03.059 25889081

[B55] Karn P. R. Kim H. D. Kang H. Sun B. K. Jin S. E. Hwang S. J. (2014). Supercritical fluid-mediated liposomes containing cyclosporin A for the treatment of dry eye syndrome in a rabbit model: Comparative study with the conventional cyclosporin A emulsion. Int. J. Nanomedicine. 9, 3791–3800. 10.2147/ijn.S65601 25143728 PMC4134020

[B56] Kaur I. P. Rana C. Singh H. (2008). Development of effective ocular preparations of antifungal agents. J. Ocul. Pharmacol. Ther. 24 (5), 481–494. 10.1089/jop.2008.0031 18788998

[B57] Kaur Jaskiran Mishra V. Singh S. K. Gulati M. Kapoor B. Kumar D. (2021). Harnessing amphiphilic polymeric micelles for diagnostic and therapeutic applications: Breakthroughs and bottlenecks. J. Control. Release 334, 64–95. 10.1016/j.jconrel.2021.04.014 33887283

[B58] Ke X. Ng V. W. Ono R. J. Chan J. M. Krishnamurthy S. Wang Y. (2014). Role of non-covalent and covalent interactions in cargo loading capacity and stability of polymeric micelles. J. Control. Release. 193, 9–26. 10.1016/j.jconrel.2014.06.061 25037018

[B59] Kikuchi A. Okano T. (2002). Intelligent thermoresponsive polymeric stationary phases for aqueous chromatography of biological compounds. Prog. Polym. Sci. 27 (6), 1165–1193. 10.1016/S0079-6700(02)00013-8

[B60] Kim D. Maharjan P. Jin M. Park T. Maharjan A. Amatya R. (2019). Potential albumin-based antioxidant nanoformulations for ocular protection against oxidative stress. Pharmaceutics 11 (7), 297. 10.3390/pharmaceutics11070297 31248013 PMC6680573

[B61] Kim K. T. Lee H. Kim J. Y. Chae J. B. Hyung S. Kim D. Y. (2020). Association between early anatomic response and intraocular pressure change after intravitreal dexamethasone implant: An optical coherence tomography study. J. Clin. Med. 9 (9), 2692. 10.3390/jcm9092692 32825291 PMC7564688

[B62] Kumar R. Sinha V. R. (2017). Evaluation of ocular irritation and bioavailability of voriconazole loaded microemulsion. Curr. Drug. Deliv. 14 (5), 718–724. 10.2174/1567201813666160816105905 27538459

[B63] Kwon S. Singh R. K. Perez R. A. Abou Neel E. A. Kim H. W. Chrzanowski W. (2013). Silica-based mesoporous nanoparticles for controlled drug delivery. J. Tissue. Eng. 4, 204173141350335. 10.1177/2041731413503357 PMC3764983 24020012

[B64] Lakhani P. Patil A. Majumdar S. (2018). Recent advances in topical nano drug-delivery systems for the anterior ocular segment. Ther. Deliv. 9 (2), 137–153. 10.4155/tde-2017-0088 29325511 PMC6367714

[B65] Lavasanifar A. Samuel J. Kwon G. S. (2002). Poly(ethylene oxide)-block-poly(l-amino acid) micelles for drug delivery. Adv. Drug. Deliv. Rev. 54 (2), 169–190. 10.1016/S0169-409X(02)00015-7 11897144

[B66] Li C. Chen R. Xu M. Qiao J. Yan L. Guo X. D. (2018). Hyaluronic acid modified MPEG-b-PAE block copolymer aqueous micelles for efficient ophthalmic drug delivery of hydrophobic genistein. Drug. Deliv. 25 (1), 1258–1265. 10.1080/10717544.2018.1474972 29847210 PMC6058726

[B67] Li J. Li Z. Liang Z. Han L. Feng H. He S. (2018). Fabrication of a drug delivery system that enhances antifungal drug corneal penetration. Drug. Deliv. 25 (1), 938–949. 10.1080/10717544.2018.1461278 29658325 PMC6058611

[B68] Li J. Xue Y. Tian J. Liu Z. Zhuang A. Gu P. (2020). Fluorinated-functionalized hyaluronic acid nanoparticles for enhanced photodynamic therapy of ocular choroidal melanoma by ameliorating hypoxia. Carbohydr. Polym. 237, 116119. 10.1016/j.carbpol.2020.116119 32241431

[B69] Li X. Fang J. Xin M. Li Q. Wang J. Yang H. (2021). Rebaudioside A/TPGS mixed nanomicelles as promising nanocarriers for nimodipine ocular delivery. Drug. Deliv. Transl. Res. 11 (3), 1119–1132. 10.1007/s13346-020-00834-0 32783152

[B70] Li Xianglian Liu Hui Yu Ailing Lin Dan Bao Zhishu Wang Yuqin (2021). Bioinspired self-assembly supramolecular hydrogel for ocular drug delivery. Chin. Chem. Lett. 32 (12), 3936–3939. 10.1016/j.cclet.2021.03.037

[B71] Li X. Zhang Z. Li J. Sun S. Weng Y. Chen H. (2012). Diclofenac/biodegradable polymer micelles for ocular applications. Nanoscale 4 (15), 4667–4673. 10.1039/c2nr30924f 22732776

[B72] Li Z. Liu M. Ke L. Wang L. J. Wu C. Li C. (2021). Flexible polymeric nanosized micelles for ophthalmic drug delivery: Research progress in the last three years. Nanoscale Adv. 3 (18), 5240–5254. 10.1039/d1na00596k 36132623 PMC9417891

[B73] Li Z. Wu X. Li J. Yao L. Sun L. Shi Y. (2012). Antitumor activity of celastrol nanoparticles in a xenograft retinoblastoma tumor model. Int. J. Nanomedicine 7, 2389–2398. 10.2147/ijn.S29945 22661892 PMC3357982

[B74] Lim C. Kim D. W. Sim T. Ngoc H. H. Lee J. W. Lee E. (2016). Preparation and characterization of a lutein loading nanoemulsion system for ophthalmic eye drops. J. Drug. Deliv. Sci. Tec. 36, 168–174. 10.1016/j.jddst.2016.10.009

[B75] Liu S. Jones L. Gu F. X. (2012). Nanomaterials for ocular drug delivery. Macromol. Biosci. 12 (5), 608–620. 10.1002/mabi.201100419 22508445

[B76] Liu X. Zhang H. Cheng R. Gu Y. Yin Y. Sun Z. (2018). An immunological electrospun scaffold for tumor cell killing and healthy tissue regeneration. Mat. Horiz. 5 (6), 1082–1091. 10.1039/c8mh00704g PMC6333278 30713696

[B77] Lu C. Yoganathan R. B. Kociolek M. Allen C. (2013). Hydrogel containing silica shell cross-linked micelles for ocular drug delivery. J. Pharm. Sci. 102 (2), 627–637. 10.1002/jps.23390 23203974

[B78] Luo X. Cui X. T. (2009). Sponge-like nanostructured conducting polymers for electrically controlled drug release. Electrochem Commun. 11 (10), 1956–1959. 10.1016/j.elecom.2009.08.027 20160915 PMC2770182

[B79] Maulvi F. A. Soni T. G. Shah D. O. (2016). A review on therapeutic contact lenses for ocular drug delivery. Drug. Deliv. 23 (8), 3017–3026. 10.3109/10717544.2016.1138342 26821766

[B80] Maysinger D. Lovrić J. Eisenberg A. Savić R. (2007). Fate of micelles and quantum dots in cells. Eur. J. Pharm. Biopharm. 65 (3), 270–281. 10.1016/j.ejpb.2006.08.011 17027243

[B81] Mehra N. Aqil M. Sultana Y. (2021). A grafted copolymer-based nanomicelles for topical ocular delivery of everolimus: Formulation, characterization, *ex-vivo* permeation, *in-vitro* ocular toxicity, and stability study. Eur. J. Pharm. Sci. 159, 105735. 10.1016/j.ejps.2021.105735 33516808

[B82] Mun J. Mok J. W. Jeong S. Cho S. Choun-Ki J. Hahn S. K. (2019). Drug-eluting contact lens containing cyclosporine-loaded cholesterol-hyaluronate micelles for dry eye syndrome. Rsc. Adv. 9 (29), 16578–16585. 10.1039/C9RA02858G 35516366 PMC9064448

[B83] Obaidat R. Amani D. Kwiak A. Hamed R. (2022). Development of combined therapy of metronidazole and ibuprofen using *in situ* microgels for the treatment of periodontitis. J. Drug. Deliv. Sci. Tec. 71, 103314. 10.1016/j.jddst.2022.103314

[B84] Occhiutto M. L. Freitas F. R. Maranhao R. C. Costa V. P. (2012). Breakdown of the blood-ocular barrier as a strategy for the systemic use of nanosystems. Pharmaceutics 4 (2), 252–275. 10.3390/pharmaceutics4020252 24300231 PMC3834913

[B85] Opanasopit P. Yokoyama M. Watanabe M. Kawano K. Maitani Y. Okano T. (2004). Block copolymer design for camptothecin incorporation into polymeric micelles for passive tumor targeting. Pharm. Res. 21 (11), 2001–2008. 10.1023/b:pham.0000048190.53439.eb 15587921

[B86] Osouli M. Abdollahizad E. Alavi S. Mahboubi A. Abbasian Z. Haeri A. (2023). Biocompatible phospholipid-based mixed micelles for posaconazole ocular delivery: Development, characterization, and in - vitro antifungal activity. J. Biomater. Appl. 37 (6), 969–978. 10.1177/08853282221141962 36424544

[B87] Ozturk M. B. Popa M. Rata D. M. Cadinoiu A. N. Parfait F. Delaite C. (2022). Drug-loaded polymeric micelles based on smart biocompatible graft copolymers with potential applications for the treatment of glaucoma. Int. J. Mol. Sci. 23 (16), 9382. 10.3390/ijms23169382 36012646 PMC9409108

[B88] Packhaeuser C. B. Schnieders J. Oster C. G. Kissel T. (2004). *In situ* forming parenteral drug delivery systems: An overview. Eur. J. Pharm. Biopharm. 58 (2), 445–455. 10.1016/j.ejpb.2004.03.003 15296966

[B89] Patel M. Saha N. Patel S. Ahlawat P. Dharamsi A. Patel A. (2022). Development of bromfenac sodium loaded pluronic nanomicelles: Characterization and corneal permeation study. Recent Adv. Drug. Deliv. Formul. 16 (1), 68–78. 10.2174/2667387816666220128123737 35088685

[B90] Pescina S. Sonvico F. Clementino A. Padula C. Santi P. Nicoli S. (2021). Preliminary investigation on simvastatin-loaded polymeric micelles in view of the treatment of the back of the eye. Pharmaceutics 13 (6), 855. 10.3390/pharmaceutics13060855 34207544 PMC8230077

[B91] Prajna N. V. Krishnan T. Rajaraman R. Patel S. Shah R. Srinivasan M. (2017). Adjunctive oral voriconazole treatment of Fusarium keratitis: A secondary analysis from the mycotic ulcer treatment trial II. JAMA. Ophthalmol. 135 (6), 520–525. 10.1001/jamaophthalmol.2017.0616 28426856 PMC5847083

[B92] Prosperi-Porta G. Kedzior S. Muirhead B. Sheardown H. (2016). Phenylboronic-acid-based polymeric micelles for mucoadhesive anterior segment ocular drug delivery. Biomacromolecules 17 (4), 1449–1457. 10.1021/acs.biomac.6b00054 26963738

[B93] Ramanavičius A. Kaušaitė A. Ramanavičienė A. (2005). Polypyrrole-coated glucose oxidase nanoparticles for biosensor design. Sens. Actuat. B-Chem. 111-112, 532–539. 10.1016/j.snb.2005.03.038

[B94] Ricca A. M. Morshedi R. G. Wirostko B. M. (2015). High intraocular pressure following anti-vascular endothelial growth factor therapy: Proposed pathophysiology due to altered nitric oxide metabolism. J. Ocul. Pharmacol. Ther. 31 (1), 2–10. 10.1089/jop.2014.0062 25369256

[B95] Rodríguez V. Navarro M. G. Villanueva L. R. (2016). Dendrimers as a promising tool in ocular therapeutics: Latest advances and perspectives. Int. J. Pharm. 511 (1), 359–366. 10.1016/j.ijpharm.2016.07.031 27436708

[B96] Rupenthal I. D. Green C. R. Alany R. G. (2011). Comparison of ion-activated *in situ* gelling systems for ocular drug delivery. Part 2: Precorneal retention and *in vivo* pharmacodynamic study. Int. J. Pharm. 411 (1-2), 78–85. 10.1016/j.ijpharm.2011.03.043 21453763

[B97] Safwat M. A. Mansour H. F. Hussein A. K. Abdelwahab S. Soliman G. M. (2020). Polymeric micelles for the ocular delivery of triamcinolone acetonide: Preparation and *in vivo* evaluation in a rabbit ocular inflammatory model. Drug. Deliv. 27 (1), 1115–1124. 10.1080/10717544.2020.1797241 32720545 PMC7470058

[B98] Sai N. Dong X. Huang P. You L. Yang C. Liu Y. (2019). A novel gel-forming solution based on PEG-DSPE/Solutol HS 15 mixed micelles and gellan gum for ophthalmic delivery of curcumin. Molecules 25 (1), 81. 10.3390/molecules25010081 31878332 PMC6983186

[B99] Salatin S. Barar J. Barzegar-Jalali M. Adibkia K. Milani M. A. Jelvehgari M. (2016). Hydrogel nanoparticles and nanocomposites for nasal drug/vaccine delivery. Arch. Pharm. Res. 39 (9), 1181–1192. 10.1007/s12272-016-0782-0 27352214

[B100] Salatin S. (2018). Nanoparticles as potential tools for improved antioxidant enzyme delivery. J. Adv. Chem. Pharm. Mater. 1 (3), 65–66. http://advchempharm.ir/journal/index.php/JACPM/article/view/47.

[B101] Saydam M. Cheng W. Palmer N. Tierney R. Francis R. MacLellan-Gibson K. (2017). Nano-sized Soluplus® polymeric micelles enhance the induction of tetanus toxin neutralising antibody response following transcutaneous immunisation with tetanus toxoid. Vaccine 35 (18), 2489–2495. 10.1016/j.vaccine.2017.03.012 28325477

[B102] Scholz M. Doerr H. W. Cinatl J. (2003). Human cytomegalovirus retinitis: Pathogenicity, immune evasion and persistence. Trends Microbiol. 11 (4), 171–178. 10.1016/s0966-842x(03)00066-0 12706995

[B103] Schultz C. (2014). Safety and efficacy of cyclosporine in the treatment of chronic dry eye. Ophthalmol. Eye. Dis. 6, OED.S16067–42. 10.4137/oed.S16067 PMC4076204 25002818

[B104] Shi H. Huai S. Wei H. Xu Y. Lei L. Chen H. (2023). Dissolvable hybrid microneedle patch for efficient delivery of curcumin to reduce intraocular inflammation. Int. J. Pharm. 643, 123205. 10.1016/j.ijpharm.2023.123205 37422141

[B105] Shi H. Zhu Y. Xing C. Li S. Bao Z. Lei L. (2022). An injectable thermosensitive hydrogel for dual delivery of diclofenac and Avastin® to effectively suppress inflammatory corneal neovascularization. Int. J. Pharm. 625, 122081. 10.1016/j.ijpharm.2022.122081 35934166

[B106] Shi S. Peng F. Zheng Q. Zeng L. Chen H. Li X. (2019). Micelle-solubilized axitinib for ocular administration in anti-neovascularization. Int. J. Pharm. 560, 19–26. 10.1016/j.ijpharm.2019.01.051 30710659

[B107] Shi W. Xie L. Wang S. (2002). Prolongation of corneal allograft survival in mice with a cyclosporine drug delivery system implant. Zhonghua Yan Ke Za Zhi 38 (8), 502–505. 12410993

[B108] Shirizadeh B. Maghsoodi M. Alami-Milani M. Salatin S. Jelvehgari M. (2017). Tailored hydrogel microbeads of sodium carboxymethylcellulose as a carrier to deliver mefenamic acid: Transmucosal administration. Jundishapur J. Nat. Pharm. Prod. 10.5812/jjnpp.65324

[B109] Shubber S. Vllasaliu D. Rauch C. Jordan F. Illum L. Stolnik S. (2015). Mechanism of mucosal permeability enhancement of CriticalSorb® (Solutol® HS15) investigated *in vitro* in cell cultures. Pharm. Res. 32 (2), 516–527. 10.1007/s11095-014-1481-5 25190006 PMC4300420

[B110] Sipos B. Budai-Szűcs M. Kókai D. Orosz L. Burián K. Csorba A. (2022). Erythromycin-loaded polymeric micelles: *In situ* gel development, *in vitro* and *ex vivo* ocular investigations. Eur. J. Pharm. Biopharm. 180, 81–90. 10.1016/j.ejpb.2022.09.023 36183927

[B111] Sun C. Li W. Ma P. Li Y. Zhu Y. Zhang H. (2020). Development of TPGS/F127/F68 mixed polymeric micelles: Enhanced oral bioavailability and hepatoprotection of syringic acid against carbon tetrachloride-induced hepatotoxicity. Food. Chem. Toxicol. 137, 111126. 10.1016/j.fct.2020.111126 31954714

[B112] Sun F. Zheng Z. Lan J. Li X. Li M. Song K. (2019). New micelle myricetin formulation for ocular delivery: Improved stability, solubility, and ocular anti-inflammatory treatment. Drug. Deliv. 26 (1), 575–585. 10.1080/10717544.2019.1622608 31172843 PMC6567238

[B113] Sun X. Sheng Y. Li K. Sai S. Feng J. Li Y. (2022). Mucoadhesive phenylboronic acid conjugated chitosan oligosaccharide-vitamin E copolymer for topical ocular delivery of voriconazole: Synthesis, *in vitro*/vivo evaluation, and mechanism. Acta. Biomater. 138, 193–207. 10.1016/j.actbio.2021.10.047 34757228

[B114] Terreni E. Chetoni P. Burgalassi S. Tampucci S. Zucchetti E. Chipala E. (2021). A hybrid ocular delivery system of cyclosporine-A comprising nanomicelle-laden polymeric inserts with improved efficacy and tolerability. Biomater. Sci. 9 (24), 8235–8248. 10.1039/d1bm01453f 34753159

[B115] Tsujinaka H. Fu J. Shen J. Yu Y. Hafiz Z. Kays J. (2020). Sustained treatment of retinal vascular diseases with self-aggregating sunitinib microparticles. Nat. Commun. 11 (1), 694. 10.1038/s41467-020-14340-x 32019921 PMC7000758

[B116] Upadhayay P. Kumar M. Pathak K. (2016). Norfloxacin loaded pH triggered nanoparticulate *in-situ* gel for extraocular bacterial infections: Optimization, ocular irritancy and corneal toxicity. Iran. J. Pharm. Re.s 15 (1), 3–22. PMC4986130 27610144

[B117] Uppalapati D. Sharma M. Aqrawe Z. Coutinho F. Rupenthal I. D. Boyd B. J. (2018). Micelle directed chemical polymerization of polypyrrole particles for the electrically triggered release of dexamethasone base and dexamethasone phosphate. Int. J. Pharm. 543 (1-2), 38–45. 10.1016/j.ijpharm.2018.03.039 29581065

[B118] Varela-Garcia A. Concheiro A. Alvarez-Lorenzo C. (2018). Soluplus micelles for acyclovir ocular delivery: Formulation and cornea and sclera permeability. Int. J. Pharm. 552 (1), 39–47. 10.1016/j.ijpharm.2018.09.053 30253214

[B119] Wang B. Liu H. Wang Z. Shi S. Nan K. Xu Q. (2017). A self-defensive antibacterial coating acting through the bacteria-triggered release of a hydrophobic antibiotic from layer-by-layer films. J. Mat. Chem. B 5 (7), 1498–1506. 10.1039/c6tb02614a 32264640

[B120] Wang D. Luo M. Huang B. Gao W. Jiang Y. LiNan K. (2020). Localized co-delivery of CNTF and FK506 using a thermosensitive hydrogel for retina ganglion cells protection after traumatic optic nerve injury. Drug. Deliv. 27 (1), 556–564. 10.1080/10717544.2020.1748759 32351142 PMC7241497

[B121] Wu Y. Yao J. Zhou J. Dahmani F. Z. (2013). Enhanced and sustained topical ocular delivery of cyclosporine A in thermosensitive hyaluronic acid-based *in situ* forming microgels. Int. J. Nanomedicine. 8, 3587–3601. 10.2147/ijn.S47665 24092975 PMC3788692

[B122] Xu J. Chen P. Zhao G. Wei S. Li Q. Guo C. (2022). Copolymer micelle-administered melatonin ameliorates hyperosmolarity-induced ocular surface damage through regulating PINK1-mediated mitophagy. Curr. Eye. Res. 47 (5), 688–703. 10.1080/02713683.2021.2022163 35179400

[B123] Xu J. Ge Y. Bu R. Zhang A. Feng S. Wang J. (2019). Co-delivery of latanoprost and timolol from micelles-laden contact lenses for the treatment of glaucoma. J. Control. Release 305, 18–28. 10.1016/j.jconrel.2019.05.025 31103677

[B124] Xu J. Xue Y. Hu G. Lin T. Gou J. Yin T. (2018). A comprehensive review on contact lens for ophthalmic drug delivery. J. Control. Release. 281, 97–118. 10.1016/j.jconrel.2018.05.020 29782944

[B125] Xu L. Qiu W. X. Liu W. L. Zhang C. Zou M. Z. Sun Y. X. (2019). PLA-PEG micelles loaded with a classic vasodilator for oxidative cataract prevention. *Acs. Biomater. Sci. Eng*. 5 (2), 407–412. 10.1021/acsbiomaterials.8b01089 33405805

[B126] Xu L. Wang Y. Nguyen V. T. Chen J. (2016). Effects of topical antibiotic prophylaxis on wound healing after flapless implant surgery: A pilot study. J. Periodontol. 87 (3), 275–280. 10.1902/jop.2015.150464 26537369

[B127] Xu T. Wang B. Liu H. Wang H. Yin P. Dong W. (2020). Prevalence and causes of vision loss in China from 1990 to 2019: Findings from the global burden of disease study 2019. Lancet. Public. Health 5, e682–e691. 10.1016/S2468-2667(20)30254-1 33271081

[B128] Xu X. Weng Y. Xu L. Chen H. (2013). Sustained release of Avastin® from polysaccharides cross-linked hydrogels for ocular drug delivery. Int. J. Bio.l Macromol. 60, 272–276. 10.1016/j.ijbiomac.2013.05.034 23748006

[B129] Yawn B. P. Wollan P. C. St Sauver J. L. Butterfield L. C. (2013). Herpes zoster eye complications: Rates and trends. Mayo. Clin. Proc. 88 (6), 562–570. 10.1016/j.mayocp.2013.03.014 23664666 PMC3788821

[B130] Younes N. F. Abdel-Halim S. A. Elassasy A. I. (2018). Solutol HS15 based binary mixed micelles with penetration enhancers for augmented corneal delivery of sertaconazole nitrate: Optimization, *in vitro*, *ex vivo* and *in vivo* characterization. Drug. Deliv. 25 (1), 1706–1717. 10.1080/10717544.2018.1497107 30442039 PMC6249589

[B131] Yu K. Cao W. Xu B. Shi W. Zhao Z. (2022). Bolld-ocular barrier and status of systemic administration of drugs in ocular diseases. Clin. Medicat. J. 20 (09), 24–27.

[B132] Yu W. Shevtsov M. Chen X. Gao H. (2020). Advances in aggregatable nanoparticles for tumor-targeted drug delivery. Chin. Chem. Lett. 31, 1366–1374. 10.1016/j.cclet.2020.02.036

[B133] Yu Y. Chen D. Li Y. Yang W. Tu J. Shen Y. (2018). Improving the topical ocular pharmacokinetics of lyophilized cyclosporine A-loaded micelles: Formulation, *in vitro* and *in vivo* studies. Drug. Deliv. 25 (1), 888–899. 10.1080/10717544.2018.1458923 29631468 PMC6058700

[B134] Zawadzka M. Dabrowski M. Gozdz A. Szadujkis B. Sliwa M. Lipko M. (2012). Early steps of microglial activation are directly affected by neuroprotectant FK506 in both *in vitro* inflammation and in rat model of stroke. J. Mol. Med. Berl. 90 (12), 1459–1471. 10.1007/s00109-012-0925-9 22806180 PMC3506835

[B135] Zhang C. Liu Z. Wang H. Feng X. He C. (2017). Novel anti-biofouling soft contact lens: l-Cysteine conjugated amphiphilic conetworks via RAFT and thiol-ene click chemistry. Macromol. Biosci. 17 (7), 1600444. 10.1002/mabi.201600444 28251809

[B136] Zhang S. Cui D. Xu J. Wang J. Wei Q. Xiong S. (2020). Bile acid transporter mediated STC/Soluplus self-assembled hybrid nanoparticles for enhancing the oral drug bioavailability. Int. J. Pharm. 579, 119120. 10.1016/j.ijpharm.2020.119120 32035254

[B137] Zhang X. Wei D. Xu Y. Zhu Q. (2021). Hyaluronic acid in ocular drug delivery. Carbohydr. Polym. 264, 118006. 10.1016/j.carbpol.2021.118006 33910737

[B138] Zhang Y. Li G. Zhang X. Lin L. (2022). ROS-scavenging glyco-nanoplatform for synergistic antibacterial and wound-healing therapy of bacterial keratitis. J. Mat. Chem. B 10, 4575–4587. 10.1039/D2TB00667G 35639464

[B139] Zhang Y. Wang C. Yang W. Shen X. Fu S. (2005). The advance of polymeric micelles used as drug delivery. Polym. Bull. (02), 42–46.

[B140] Zhao L. Wang H. Du X. (2021). The therapeutic use of quercetin in ophthalmology: Recent applications. Biomed. Pharmacother. 137, 111371. 10.1016/j.biopha.2021.111371 33561647

[B141] Zhao X. Seah I. Xue K. Wong W. Tan Q. S. W. Ma X. (2022). Antiangiogenic nanomicelles for the topical delivery of aflibercept to treat retinal neovascular disease. Adv. Mat. 34 (25), e2108360. 10.1002/adma.202108360 34726299

[B142] Zheng X. Xie J. Zhang X. Sun W. Zhao H. Li Y. (2021). An overview of polymeric nanomicelles in clinical trials and on the market. Chin. Chem. Lett. 32 (1), 243–257. 10.1016/j.cclet.2020.11.029

[B143] Zhou T. Zhu L. Xia H. He J. Liu S. He S. (2017). Micelle carriers based on macrogol 15 hydroxystearate for ocular delivery of terbinafine hydrochloride: *In vitro* characterization and *in vivo* permeation. Eur. J. Pharm. Sci. 109, 288–296. 10.1016/j.ejps.2017.08.020 28823856

[B144] Zhu L. Zhu H. (2014). Ocular herpes: The pathophysiology, management and treatment of herpetic eye diseases. Virol. Sin. 29 (6), 327–342. 10.1007/s12250-014-3539-2 25547680 PMC8206444

[B145] Zhuk I. Jariwala F. Attygalle A. Wu Y. Libera M. Sukhishvili S. (2014). Self-defensive layer-by-layer films with bacteria-triggered antibiotic release. Acs. Nano. 8, 7733–7745. 10.1021/nn500674g 25093948

